# Cytospyrone and Cytospomarin: Two New Polyketides Isolated from Mangrove Endophytic Fungus, *Cytospora* sp. †

**DOI:** 10.3390/molecules25184224

**Published:** 2020-09-15

**Authors:** Chengwen Wei, Qin Deng, Mengyu Sun, Jing Xu

**Affiliations:** 1 Key Laboratory of Advanced Materials of Tropical Island Resources of Ministry of Education, School of Chemical Engineering and Technology, Hainan University, Haikou 570228, China; chengwen121412@163.com (C.W.); sunmengyu0305@163.com (M.S.); 2 College of Horticulture and Landscape, Hainan University, Haikou 570228, China; dengqin_e@yeah.net

**Keywords:** *Cytospora* sp., polyketides, structure identification, antimicrobial activity, cytotoxicity

## Abstract

Two new polyketides, cytospyrone (**1**), cytospomarin (**2**), together with three known metabolites dimethoxyphtalide (**3**), integracin A (**4**) and integracin B (**5**), were isolated from the culture of *Cytospora* sp. from the Chinese mangrove *Ceriops tagal*. Their structures were elucidated by extensive spectroscopic analyses and time dependent density functional theory (TDDFT), calculation of electronic circular dichroism (ECD) and optical rotation (OR) data. Compound **2** displayed weak inhibitory activity against *Escherichia coli* GIM1.201 (minimum inhibitory concentration (MIC) value of 0.35 mM). Compounds **4** and **5** displayed significant cytotoxicity against human cancer cell line HepG2 (IC_50_ values of 5.98 ±  0.12 µM and 9.97 ± 0.06 µM, respectively), more potent than the positive control 5-fluorouracil (IC_50_ value of 43.50 ± 3.69 µM).

## 1. Introduction

Mangrove endophytic fungi are attracting considerable attention from natural product chemists and biologists alike and a great deal of structurally diverse natural products with unusual biological activity have been discovered recently [1,2,3]. Fungal genera *Alternaria*, *Aspergillus*, *Cladosporium*, *Colletotrichum*, *Fusarium*, *Paecilomyces*, *Penicillium*, *Pestalotiopsis*, *Phoma*, *Phomopsis*, *Phyllosticta*, and *Trichoderma* are considered as the predominant producer of the most notable bioactive secondary metabolites [4,5,6]. However, due to the high rediscovery of previously known compounds and scaffolds from these well-investigated organisms, neglected fungal species that are poorly studied might be an alternative source for the discovery of new bioactive compounds.

**Figure 1:**
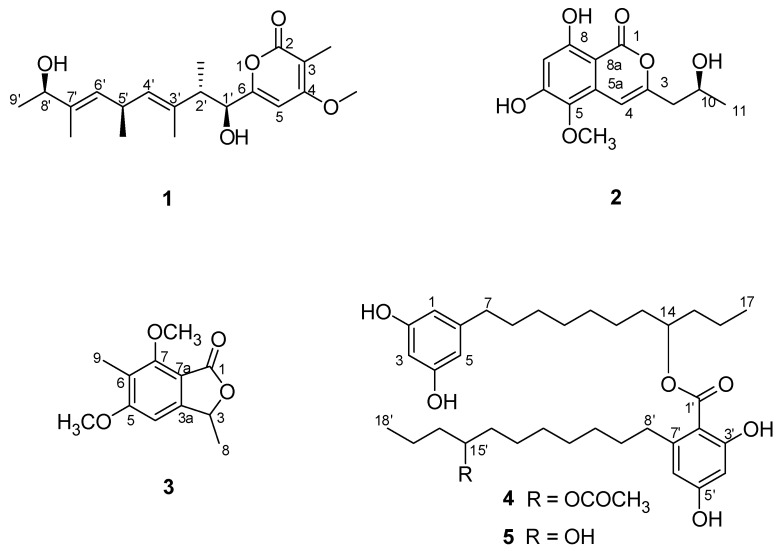
Structures of compounds **1–5.**

During our ongoing screening for biologically active secondary metabolites from mangrove endophytic fungi [7,8,9,10], we recently investigated *Cytospora* sp., an endophytic fungus derived from the hypocotyls of Chinese mangrove *Ceriops tagal*, resulting in the isolation of a new antimicrobial biscyclic sesquiterpene, seiricardine D, together with eight known metabolites [11]. Further study of the aforementioned *Cytospora* sp. afforded two new polyketides, cytospyrone (**1**), cytospomarin (**2**), together with three known metabolites dimethoxyphtalide (**3**) [12], integracin A (**4**) [13], and integracin B (**5**) (Figure 1) [13].

## 2. Results and Discussion

### 2.1. Isolation and Structure Elucidation

**Figure 2:**
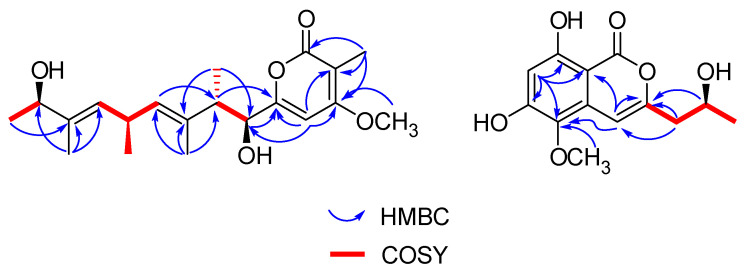
Key HMBC, ^1^H-^1^H COSY correlations of **1** and **2.**

**Figure 3:**
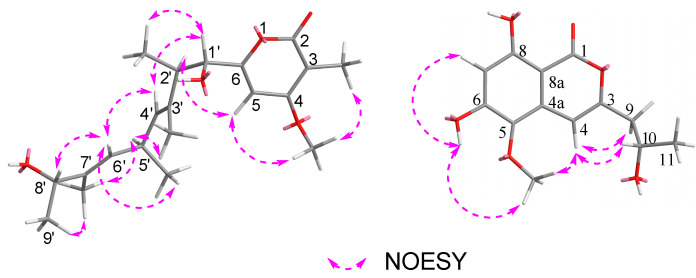
Key NOESY correlations of **1** and **2.**

**Figure 4:**
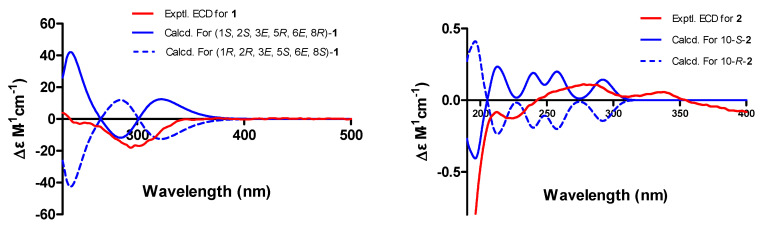
Experimental and calculated electronic circular dichroism (ECD) spectra of **1** and **2.**

Cytospyrone A (**1**) was isolated as a white amorphous powder, and has the molecular formula C_20_H_30_O_5_ established by high resolution electrospray ionization mass spectroscopy (HR-ESI-MS) at *m*/*z* 373.1988 [M + Na]^+^ (calculated for 373.1991), indicating six degrees of unsaturation. The ^1^H and ^13^C nuclear magnetic resonance (NMR) data of **1** in association with distortionless enhancement by polarization transfer (DEPT) and heteronuclear single quantum coherence (HSQC) spectrum suggested the presence of six upfield methyls [*δ*_H_ 1.03, (d, *J =* 6.7), *δ*_C_ 21.7, q, 5′-CH_3_; 1.07, (d, *J =* 7.0), *δ*_C_ 16.2, q, 2′-CH_3_; 1.18, (d, *J =* 6.4), *δ*_C_ 21.9, q, 9′-CH_3_; 1.65, s, *δ*_C_ 11.8, q, 7′-CH_3_; 1.67, s, *δ*_C_ 10.9, q, 3′-CH_3_; 1.85, s, *δ*_C_ 8.4, q, 2-CH_3_], a methoxy group (*δ*_H_ 3.94, s, *δ*_C_ 57.3, q, 4-OCH_3_), three olefinic signals [*δ*_H_ 5.26, (d, *J =* 9.0), *δ*_C_ 130.0, d, CH-6′; *δ*_H_ 5.32, (d, *J =* 9.1), *δ*_C_ 130.0, d, CH-4′; *δ*_H_ 6.47, s, *δ*_C_ 97.5, d, CH-5], four methines [2.79, m, *δ*_C_ 44.1, d, 2′-CH; 3.33-3.42, m, *δ*_C_ 32.3, d, 5′-CH; 4.08, (d, *J =* 9.6), *δ*_C_ 80.7, d, 1′-CH; 4.10, (q, *J =* 6.4), *δ*_C_ 73.9, d, 8′-CH], and six quaternary carbons (*δ*_C_ 101.5, 134.2, 138.1, 167.8 168.5, 169.1). Two substructures, an α-pyrone moiety and a linear aliphatic chain, were assigned by analyses of detailed interpretation of ^1^H-^1^H correlation spectroscopy (COSY) and heteronuclear multiple bond correlation (HMBC) correlations (Figure 2). The α-pyrone moiety was established by HMBC correlations from 3-CH_3_ to carbons C-2, C-3 and C-4, and from H-5 to C-4 and C-6. Presence of the methoxy group at C-4 was corroborated from the HMBC correlation of its protons (*δ*_H_ 3.94, s) with C-4 (*δ*_C_ 169.1) and nuclear overhauser effect spectroscopy (NOESY) correlations between 3-CH_3_/4-OCH_3_ and H-5/4-OCH_3_. A linear aliphatic chain with a thirteen carbon unit was determined by the interpretation of ^1^H–^1^H COSY and HMBC spectroscopic data. The COSY cross-peaks [H-1′/H-2′/2′-CH_3_, H-3′/H-4′/4′-CH_3_/H-5′, H-8′/H-9′] and HMBC correlations from 2′-CH_3_ to carbons C-1′, C-3′, from 3′-CH_3_ to carbons C-2′, C-3′ and C-4′, from 7′-CH_3_ to carbons C-6′, C-7′and C-8′, and from H-9′ to C-7′ permitted the construction of a linear aliphatic chain. The above two structural fragments were finally connected to construct the planar structure of compound **1** from the key HMBC correlations from H-1′ and H-2′ to C-6, from H-5 to C-2′. The relative configuration of **1** was deduced by analysis of the NOESY data. The NOESY correlations (Figure 3) between H-1′/2′-CH_3_, 2′-CH_3_/ H-4′, H-2′/3′-CH_3_, H-4′/ H-5′, H-5′/H-6′, 7′-CH_3_/H-8′ and H-6′/H-8′ suggested that they were positioned on the same face and both ∆^4′^ and ∆^6′^ double bonds were assigned to the *E*-configuration. In addition, the correlations of H-1′/3′-CH_3_, 3′-CH_3_/5′-CH_3_ and 5′-CH_3_/ 7′-CH_3_ revealed they were positioned on the other face. To define its absolute configuration, the time dependent density functional theory—(TDDFT)-calculated electronic circular dichroism (ECD) spectrum at the B3LYP/6-31+g (d, p) level was compared with the experimental circular dichroism (CD) spectrum (Figure 4). Therefore, the structure of **1** was established as 6-((1*S*, 2*S*, 3*E*, 5*R*, 6*E*, 8*R*)-1,8-dihydroxy-2,3,5,7-tetramethylnona-3,6-dienyl)-4-methoxy-3-methyl-2*H*-pyran-2-one.

Cytospomarin (**2**), a yellow amorphous solid, was found to have the molecular formula C_13_H_14_O_6_, established by HR-ESI-MS (*m*/*z* 289.0674, calculated for [M + Na]^+^ 289.0688), implying seven degrees of unsaturation. The ^1^H and ^13^C NMR data of **2** indicated that six of the seven units of unsaturation could be due to an aromatic ring, a double bond, and one carbonyl group. Thus, the remaining unit of unsaturation was attributed to a ring formation. The ultraviolet (UV) absorption maxima at 247 and 318 nm suggested **2** is an isocoumarin derivative. The ^1^H and ^13^C NMR data of **2** and the DEPT and HSQC spectra revealed the presence of two methyl groups, including a methoxy group (*δ*_H_ 3.68, s, *δ*_C_ 60.4, q, 5-OCH_3_) and a terminal alkyl methyl [*δ*_H_ 1.12, (d, *J =* 6.2), *δ*_C_ 23.3, q, 11-CH_3_], a methylene (*δ*_H_ 2.50–2.57, m, *δ*_C_ 42.8, t, CH_2_-9], two olefinic methines [*δ*_H_ 6.33, s, *δ*_C_ 102.6, d, CH-7; *δ*_H_ 6.53, s, *δ*_C_ 99.9, d, CH-4], an oxymethine (*δ*_H_ 3.92–4.00, m, *δ*_C_ 64.0, d, CH-10) and seven quaternary carbons, including a carbonyl (*δ*_C_ 165.6, s, C-1). The ^1^H and ^13^C NMR and HSQC spectra of compound **2** closely resembled those of an isocoumarin compound peniisocoumarin G [14], recently isolated from another endophytic fungus, *Penicillium commune* QQF-3, obtained from fresh fruit of the mangrove plant *Kandelia candel*, indicating that **2** might have the same basic molecular framework as peniisocoumarin G. The only difference between these was replacement of the hydroxyl substituent of peniisocoumarin G by a methoxy group in **2**. The HMBC and NOESY correlations (Figure 2 and Figure 3) supported the assignments of the methoxy group 5-OCH_3_ (*δ*_H_ 3.68, s) at C-5 (*δ*_C_ 134.3). The configuration of C-10 was determined by comparison of optical rotation with the known compounds peniisocoumarin G [14] and botryosphaerin A [15]. The result indicated that compound **2** has the same positive value as those two isocoumarins. It was confirmed by comparing experimental and calculating ECD spectra and optical rotation ([α]^20^_D_= + 11.7°(c 0.3, MeOH); calculated for [α]^20^_D_ = +89.6°(MeOH)) of **2** using TDDFT (Figure 4). Accordingly, compound **2** was determined as (*S*)-6,8-dihydroxy-3-(2-hydroxypropyl)-5-methoxy-1*H*-isochromen-1-one and named as cytospomarin.

### 2.2. Bioactivities

Compounds **1**−**5** were testing the in vitro antimicrobial activity against four human pathogens *Escherichia coli* GIM1.201, methicillin-resistant *Staphylococcus aureus* GIM1.771 (MRSA), *Pseudomonas aeruginosa* GIM1.200, *Candida albicans* GIM2.169, together with three plant pathogens including *Bacillus subtilis*, *Colletotrichum gloeosporioides* and *Magnaporthe oryze*, and the results are summarized in Table 1. Compound **1**−**5** were also tested in vitro against the human cancer cell line HepG2 with 5-fluorouracil as the positive control. Among them, compound **2** exhibited weak inhibitory activity against *E. coli* GIM1.201 (minimum inhibitory concentration (MIC) = 0.35 mM) and *M. oryzae* (MIC = 1.41 mM). Compounds **4** and **5** displayed significant cytotoxicity against human cancer cell line HepG2 with IC_50_ values of 5.98 ±  0.12 μM and 9.97 ± 0.06 μM, respectively, more potent than the positive control 5-fluorouracil (IC_50_ = 43.50 ± 3.69 μM) (Table 2).

Mangrove endophytic fungi have been proved to be particularly productive with regard to the accumulation of diverse biological natural products and yielded more than 800 natural products [16]. In our previous study, over 120 different compounds, with thirty of them being new polyketides, were discovered from mangrove endophytic fungi, including pyrones [17], isocoumarins [9,17], coumarins [18], cytosporones [18] and chromones [19], etc. In the current study, two new polyketides, cytospyrone (**1**), cytospomarin (**2**), together with three known metabolites (**3**–**5**) were isolated from the rice culture of *Cytospora* sp. from Chinese mangrove *Ceriops tagal*. Structurally, **1** and pestalotiopyrones A–H [17] are pyrone derivatives, having both a methoxy group positioned at C-4 and a substituted alkyl side chain at C-6, which all proved to be devoid of significant activity in the antimicrobial and cytotoxic bioassays. Many naturally occurring isocoumarin and 3,4-dihydroisocoumarin derivatives are known to have substituents at C-3 and have been shown to possess an impressive array of biological activities [20]. Five dihydroisocoumarins isolated from *Cytospora eucalypticola* KC1636 [21] exhibited mildly antifungal and antibacterial activities towards Gram positive bacteria, which is similar to our biological finding on **2**. Compound **3** is closely related to 5-dehydroxycytosporone E previously reported from another *Cytospora* sp. TT-10, both featuring a phthalan-1-one skeleton [22]. Previously other fungal cytosporone derivatives that bear structural similarities to **3** isolated in our study had been described to be cytotoxic against several cancer cell lines in vitro [23], in contrast to the previous report, **3** showed no activity towards the bioassays we investigated. Integracins A (**4**) and B (**5**), exhibited potent (human immunodeficiency virus Type 1) HIV-1 integrase inhibitors, and were dimeric alkyl aromatic compounds isolated for the first time from *Cytonaema* sp. living in twigs of *Quercus ilex* [13]. They were later found to be produced by various organisms, such as endophytic fungi *Cytospora* sp. [22], Chinese mangrove plant *Sonneratia hainanensis* [24] and *Laguncularia racemose* [25]. Moreover, compound **4** was also reported to possess potent cytotoxicity against the tumor cell lines HepG2 and NCI-H460 with both 100% inhibitions at 25 mg/mL [24]. In this work, we found not only **4** but **5** strongly inhibited the proliferation of human cancer cell line HepG2, which was in accordance with previous reports. This highlighted the high potential of bioprospecting of polyketides from mangrove endophytic fungi *Cytospora*.

## 3. Materials and Methods

### 3.1. General Experimental Procedures

Optical rotations were measured on a WYA-2S digital Abbe refractometer (Shanghai Physico-optical Instrument Factory, Shanghai, China). UV spectra were obtained on a Shimadzu UV-2401 PC spectrophotometer (Shimadzu Corporation, Tokyo, Japan). Then, 1D and 2D NMR spectra were recorded on Bruker AV 400 NMR apparatus (Bruker Biospin Group, Karlsruhe, Germany) in CD_3_OD, DMSO-*d*_6_ or CDCl_3_ (Guangzhou Chemical Reagent Factory, Guangzhou, China). Mass spectra were obtained on a LTQ Orbitrap XL instrument (Thermo Fisher Scientific, Bremen, Germany) using peak matching. Column chromatography was performed with silica gel (200−300 mesh, Qingdao Haiyang Chemical Co., Qingdao, China) and Sephadex LH-20 (18−110 μm, Merck, Darmstadt, Germany). Semi-preparative HPLC was achieved on an Agilent 1100.

### 3.2. Fungal Material and Identification

Following standard procedures [26], endophytic fungi were isolated from fresh, healthy hypocotyls of *Ceriops tagal* (Rhizophoraceae), which were collected in October 2015 in Dong Zhai Gang-Mangrove Garden on Hainan Island, China. The fungus (strain no. JGM-9) was identified as *Cytospora* sp. (GenBank accession no. MG948459) according to DNA amplification and sequencing of the internal transcribed spacer (ITS) region. A voucher strain was deposited at one of the authors’ laboratory (J.X.).

### 3.3. Fermentation, Extraction and Isolation

The fungus was grown on solid rice medium at room temperature in 1000 mL-Erlenmeyer flasks containing 100 g rice (Jiangxi COFCO Rice Industry Co., Nanchang, China) and 100 mL-seawater. After 39 days of cultivation, the mycelia and solid rice medium were extracted with ethyl acetate (EtOAc). The extract was evaporated under reduced pressure to yield 90.0 g residue. This residue was subjected to silica gel column chromatography (CC) employing a step gradient of petroleum ether/ethyl-acetate gradient (100:0 to 0:100) to obtain four fractions (Fr. 1–Fr. 7). Promising Fr. 5 was separated by CC (silica gel; petroleum ether/ethyl acetate/acetone 5:2:2) to give four sub-fractions. Sub-Fr. 5–2 was purified with silica gel by CHCl_3_/MeOH 20:1 and reversed-phase-HPLC (MeOH/H_2_O 80:20) to yield compound **2** (15.0 mg). Sub-Fr. 5–3 was subjected to CC (Sephadex LH-20, MeOH/CHCl_3_ 1:1) and reversed-phase-HPLC (MeOH/H_2_O 70:30 to 100:0) to furnish **1** (8.1 mg), **4** (5.7 mg) and **5** (3.8 mg). Fr. 7 was subjected to CC (silica gel; gradient petroleum ether / acetone) and reversed-phase-HPLC (MeOH/H_2_O 80:20 to 100:0) to afford **3** (3.8 mg).

Cytospyrone (**1**): white amorphous powder (MeOH); [α]^20^_D_ = −23.1° (c0.05, MeOH); UV(MeOH) λ_max_ 212 nm; ^1^H NMR(400 MHz, CD_3_OD) *δ*_H_ 6.47 (1H, s, H-5), 5.32 (1H, d, *J* = 9.1 Hz, H-4′), 5.29 (1H, d, *J* = 9.0, 6′), 4.10 (1H, q, *J* = 6.4, H-8′), 4.08 (1H, d, *J* = 9.6, H-1′), 3.94 (3H, s, 4-OCH_3_), 3.33–3.42 (1H, m, H-5′), 2.79 (1H, m, H-2′), 1.85 (3H, s, 3-CH_3_), 1.67 (3H, s, 3′-CH_3_), 1.65 (3H, s, 7′-CH_3_), 1.18 (3H, d, *J* = 6.4, H-9′), 1.07 (3H, d, *J* = 7.0, 2′-CH_3_), 1.03 (3H, d, *J* = 6.7, 5′-CH_3_); ^13^C-NMR (100 MHz, CD_3_OD) *δ*_C_ 169.1 (C-4), 168.5 (C-2), 167.8 (C-6), 138.1 (C-7′), 135.3 (C-4′), 134.2 (C-3′), 130.1 (C-6′), 101.5 (C-3), 97.5 (C-5), 80.7 (C-1′), 73.9 (C-8′), 57.3 (4-OCH_3_), 44.1 (C-2′), 32.3 (C-5′), 21.9 (C-9′), 21.7 (5′-CH_3_), 16.2 (3′-CH_3_), 11.8 (7′-CH_3_), 10.8 (3′-CH_3_), 8.4 (3-CH_3_); HR-ESI-MS *m*/*z* 373.1991 [M + Na]^+^ (calculated for C_20_H_30_O_5_Na, 373.1991) (Supplementary Materials).

Cytospomarin (**2**)**:** a yellow amorphous solid (MeOH); [α]^20^_D_ = +11.7° (c 0.3, MeOH); UV(MeOH)λ_max_ 318 nm, 247 nm; ^1^H NMR (400 MHz, DMSO-*d*_6_) *δ*_H_ 10.8 (1H, br s, 6-OH), 6.53 (1H, s, H-4), 6.33 (1H, s, H-7), 3.92–4.00 (1H, m, H-10) 3.68 (3H, s, 5-OCH_3_), 2.50–2.57 (2H, m, H-9), 1.12 (3H, d, *J* = 6.2, H-11); ^13^C-NMR (100 MHz, DMSO-*d*_6_) *δ*_C_ 165.6 (C-1), 161.1 (C-8), 158.6 (C-6), 154.7 (C-3), 134.3 (C-5), 130.3 (C-5a), 102.6 (C-7), 99.9 (C-4), 95.5 (C-8a), 64.0 (C-10), 60.4 (5-OCH_3_), 42.8 (C-9), 23.3 (C-11); HRESIMS *m/z* 289.0674 [M + Na]^+^ (calculated for C_13_H_14_O_6_Na, 289.0610) (Supplementary Materials).

Dimethoxyphtalide (**3**): white amorphous powder (MeOH); [α]^20^_D_ = +123° (c 0.1, MeOH), UV(MeOH)λ_max_ 216 nm, ^1^H NMR(400 MHz, CDCl_3_) *δ*_H_ 6.55 (1H, s, H-4), 5.40 (1H, d, *J* = 6.6, H-3), 4.05 (3H, s, 7-OCH_3_), 3.92 (3H, s, 5-OCH_3_), 2.14 (3H, s, 6-CH_3_), 1.59 (3H, d, *J* = 6.6, 3-CH_3_); ^13^C-NMR (100 MHz, CDCl_3_) *δ*_C_ 168.3 (C-1), 164.2 (C-5), 157.6 (C-7), 152.9 (C-3a), 120.5 (C-6), 109.7 (C-7a), 97.9 (C-4), 76.4 (C-3), 62.1 (7-OCH_3_), 56.1 (5-OCH_3_), 29.7 (C-9), 20.7 (C-8); HRESIMS *m/z* 233.0969 [M + H]^+^ (calculated for C_12_H_15_O_4_, 233.0969). The data of ^1^H NMR and ^13^C NMR are basically consistent with those reported in literature [12].

Integracin A (**4**): yellow oil (MeOH); [α]^20^_D_ = +97.0° (c 0.1, MeOH); UV (MeOH) max 280,259,218 nm; ^1^H NMR(500 MHz, CD3OD) 6.23 (H, d, *J* = 1.8, H-6′), 6.19 (H, d, *J* = 1.8, H-4′), 6.14 (2H, d, *J*  = 1.4, H-1, H-5), 6.10 (H, br s, H-3), 5.28 (H, pent, *J* = 5.6 Hz, H-14), 4.82 (H, overlapped, H-15′), 2.81–2.85 (2H, m, H-8′), 2.45 (H, t, *J* = 8.0 Hz, H-7), 2.03 (3H, s, Me-2′′), 1.66–1.76 (4H, m, H-13, H-15), 1.54–1.57 (4H, m, H-8, H-9′), 1.40–1.42 (4H, m, H-14′, H-16′), 1.31–1.35 (20H, m, 9-H to 12-H, H-16, H-10′ to H-13′, H-17′), 0.99 (3H, t, *J* = 7.3 Hz, Me-18′), 0.94 (3H, t, *J* = 7.3, Me-17); 13C-NMR (125 MHz, CD3OD) δ 173.1 (C-1′), 172.7 (C-1′′),165.9 (C-3′), 163.5 (C-5′), 159.2(C-2, C-4), 149.0 (C-7′), 146.3 (C-6), 111.9 (CH-6′), 107.9 (CH-1, CH-5), 105.8 (C-2′), 101.9 (CH-4′), 101.0 (CH-3), 76.7 (CH-14), 75.5 (CH-15′), 37.7 a (CH2-8′), 37.4 a (CH2-15), 37.4 a (CH2-16′), 36.9 (CH2-7), 35.2 (CH2-13), 35.2 (CH2-14′), 33.6 (CH2-9′), 32.3 (CH2-8), 31.0 a (CH2-10′), 30.7 a (CH2-11′), 30.5 a (CH2-12′), 30.5 a (CH2-11), 30.4 a (CH2-10), 30.2 (CH2-9), 26.6 (CH2-13′), 26.4 (CH2-12), 21.2 (CH3-2′′), 19.9 (CH2-16), 19.6 (CH2-17′), 14.3 (CH3-18′), 14.2 (CH3-17), chemical shifts could be interchanged; ESI-MS [M + H]+ *m*/*z* 629.4 (C37H57O8). The data of 1H NMR and 13C NMR are basically consistent with those reported in literature [13].

Integracin B (**5**): yellow oil (MeOH); [α]20D = +37.8° (c 0.30, MeOH); UV (MeOH) λ_max_ 300, 264, 230 nm; 1H NMR(500 MHz, CD3OD) δ 11.98 (H, s, 3′-OH), 7.61 (H, br s, 3×-OH), 6.21 (H, d, *J* = 2.4, H-6′), 6.19 (H, d, *J* = 2.4 Hz, H-4′), 6.14 (2H, d, *J* = 2.1 , H-1, H-5), 6.10 (H, t, *J* = 2.1, H-3), 5.20 (H, pent, *J* = 5.7, H-14), 3.48–3.52 (H, m, H-15′), 2.80 (2H, td, *J* = 7.0, 2.5, H-8′), 2.40 (H, t, *J* = 7.5, H-7), 1.63–1.67 (2H, m, H-13), 1.59–1.63 (2H, m, H-15), 1.48–1.55 (4H, m, H-8, H-9′), 1.36–1.39 (4H, m, H-14′, H-16′), 1.34–1.36 (2H, m, H-17′), 1.28–1.30 (2H, m, H-16), 1.23–1.28 (20H, m, 9-H to 12-H, H-16, H-10′ to H-13′, H-17′), 0.91 (3H, t, *J* = 7.3, Me-18′), 0.88 (3H, t, *J* = 6.8, Me-17); 13C-NMR (125 MHz, CD3OD) δ 172.3 (C-1′), 165.5 (C-3′), 162.8 (C-5′), 158.4(C-2, C-4), 149.1 (C-7′), 146.1 (C-6), 111.8 (CH-6′), 107.8 (CH-1, CH-5), 105.1(C-2′), 101.6 (CH-4′), 100.6 (CH-3), 76.3 (CH-14), 71.9 (CH-15′), 40.0 (CH2-16′), 37.9 (CH2-8′), 37.6 (CH2-15), 37.2 (CH2-7), 36.6 (CH2-13), 35.0 (CH2-14′), 33.1 (CH2-9′), 31.9 (CH2-8), 30.7 a (CH2-10′), 30.5 a (CH2-11′), 30.4 a (CH2-12′), 30.1 a (CH2-11), 30.0 a (CH2-10), 29.9 (CH2-9), 26.4 (CH2-13′), 26.2 (CH2-12), 19.5 (CH2-16), 19.4 (CH2-17′), 14.4 (CH3-18′), 14.3 (CH3-17), a chemical shifts could be interchanged; ESIMS [M + H]+ *m*/*z* 587.4 (C35H55O7). The data of 1H NMR and 13C NMR are basically consistent with those reported in literature [13].

### 3.4. ECD Calculations

Monte Carlo conformational searches were carried out by means of Spartan 14 software using Merck molecular force field (MMFF). The conformers with Boltzmann population of over 1% were chosen for ECD calculations, and then the conformers were initially optimized at B3LYP/6-31g level in gas. The theoretical calculation of ECD was conducted in MeOH using time dependent density functional theory (TDDFT) at the B3LYP/6-31+g (d, p) level for all conformers of compounds **1** and **2**. ECD spectra were generated using the program SpecDis 1.6 (University of Würzburg, Würzburg, Germany) and GraphPad Prism 5 (University of California, San Diego, CA, USA) from dipole-length rotational strengths by applying Gaussian band shapes with sigma = 0.3 eV.

### 3.5. Optical Rotation (OR) Calculations

Monte Carlo conformational searches were carried out by means of Spartan 10 software using Merck molecular force field (MMFF). The conformers with Boltzmann population of over 1% were chosen for OR calculations, and then the conformers were initially optimized at B3LYP/6-31+g (d, p) level in MeOH using the conductor-like polarizable continuum model (CPCM) polarizable conductor calculation model. The theoretical calculation of OR was conducted in MeOH using time dependent density functional theory (TDDFT) at the B3LYP/6-31+g (d, p) level for all conformers of compound **2**.

### 3.6. Antimicrobial Activity

The antimicrobial activity was determined against human pathogens *Escherichia coli* GIM1.201, methicillin-resistant *Staphylococcus aureus* GIM1.771 (MRSA), *Pseudomonas aeruginosa* GIM1.200, *Candida albicans* GIM2.169 and plant pathogenic fungi *Bacillus subtilis*, *Colletotrichum gloeosporioides* and *Magnaporthe oryze* (Guangdong Microbial Culture Collection Center, China, CDMCC). The bacteria was cultured in Luria-Bertani (LB) medium (10 g of peptone, 15 g of sodium chloride, 3 g of yeast extract, and 1000 mL of distilled H_2_O) at 37 °C (160 rpm) for 24 h, and the fungi was cultured in potato dextrose broth (PDB) medium (200 g of potatoes, 20 g of dextrose, and 1000 mL of distilled H_2_O) at 28 °C (160 rpm) for 48 h. All isolated compounds were subjected to antimicrobial assays using a serial dilution technique, as previously described [26]. The minimum inhibitory concentration (MIC) was assigned to the lowest concentration that completely inhibited the growth of the indicator microorganisms. Ciprofloxacin (Guangzhou Lianghua Chemical Co., Ltd., 99.9%, Guangzhou, China) and amphotericin (American AMRESCO, 99.9%, Branded Products Group, Cochran, USA) were used as a positive control.

### 3.7. Cytotoxicity Assay

The human hepatoma cell (HepG2) cells (Cell Resource Center, Shanghai Institutes for Biological Sciences, Chinese Academy of Sciences) were grown in RPMI-1640 culture medium. Cytotoxicity against HepG2 cells was evaluated using the 3-(4,5-dimethylthiazol-2-yl)-2,5-diphenyltetrazolium bromide (MTT) (Sigma-Aldrich, Missouri, St. Louis, MO, USA) method, as described previously [8] and 5-Fluorouracil (5-FU) (Beijing Solarbio Science & Technology Co., Ltd., 99.8%) (Beijing, China) was used as the positive control.

## 4. Conclusions

In the present work, two new polyketides cytospyrone (**1**), cytospomarin (**2**) and three known metabolites (**3**−**5**) were obtained from the ethyl acetate extract of the fermented cultures of the fungus *Cytospora* sp., which was isolated from the Chinese mangrove *Ceriops tagal*. Compound **2** exhibited weak inhibitory activity against *Escherichiacoli* GIM1.201 and compounds **4** and **5** displayed potent cytotoxicity against human cancer cell line HepG2.

## Figures and Tables

**Table 1:** Antimicrobial activities of compounds **1**–**5.**

MIC (mM)							
Compound	*E. coli* GIM1.201	MRSA GIM1.771	*P. aeruginosa* GIM1.200	*C. albicans* GIM2.169	*B. subtilis*	*C. gloeosporioides*	*M. oryzae*
**1**	-	-	-	-	-	-	-
**2**	0.35	-	-	-	-	-	1.41
**3**	-	1.94	-	-	-		-
**4**	-	-	-	-	-	-	-
**5**	-	-	-	-	-	-	-
amphotericin	-	-	-	45.7 μM	-	7.6 μM	7.6 µM
ciprofloxacin	0.07	0.14	0.07	-	0.14	-	-

-: not detectable.

**Table 2:** Cytotoxicity data of compounds **1**–**5**. ^a^

Cell Line	Compound				
	**1**–**2**	**3** ^a^	**4** ^a^	**5** ^a^	**5-FU** ^a^
HepG2(IC_50_)	-	297.97 ± 17.54	5.98 ± 0.12	9.97 ± 0.06	43.50 ± 3.69

^a^ Results are expressed as IC_50_ values of mean ± SD (*n* = 3) in μM.

## Supplementary Materials

The following are available online, Figure S1. ^1^H-NMR of cytospyrone (**1**); Figure S2. ^13^C-NMR of cytospyrone (**1**); Figure S3. DEPT of cytospyrone (**1**); Figure S4. ^1^H-^1^H COSY of cytospyrone (**1**); Figure S5. HMQC of cytospyrone (**1**); Figure S6. HMBC of cytospyrone (**1**); Figure S7. ROESY of cytospyrone (**1**); Figure S8. HR-ESI-MS of cytospyrone (**1**); Figure S9 CD spectrum of cytospyrone (**1**); Figure S10. ^1^H-NMR of cytospomarin (**2**); Figure S11. ^13^C-NMR of cytospomarin (**2**); Figure S12. DEPT of cytospomarin (**2**); Figure S13. ^1^H-^1^H COSY of cytospomarin (**2**); Figure S14. HMQC of cytospomarin (**2**); Figure S15. HMBC of cytospomarin (**2**); Figure S16. ROESY of cytospomarin (**2**); Figure S17. HR-ESI-MS of cytospomarin (**2**); Figure S18. CD spectrum of cytospomarin (**2**). **Table S1.** Gibbs free energies*^a^* and equilibrium populations*^b^* of low-energy conformers of cytospyrone; Table S2. Cartesian coordinates for the low-energy reoptimized MMFF conformers of cytospyrone (**1**) at B3LYP/6-31G(d,p) level of theory in gas; **Table S3**. Gibbs free energies*^a^* and equilibrium populations*^b^* of low-energy conformers of cytospomarin (**2**); Table S4. Cartesian coordinates for the low-energy reoptimized MMFF conformers of cytospomarin (**2**) at B3LYP/6-31G(d,p) level of theory in gas; Table S5. Gibbs free energiesa and equilibrium populationsb of low-energy conformers of cytospomarin (**2**); Table S6. Cartesian coordinates for the low-energy reoptimized MMFF conformers of cytospomarin (**2**) at B3LYP/6-31+G(d,p) level of theory in CH_3_OH.
