# Supplementary material for: Cytospyrone and Cytospomarin: Two New Polyketides Isolated from Mangrove Endophytic Fungus, Cytospora sp
Source: Molecules. 2020 Sep 15;25(18):4224. doi: 10.3390/molecules25184224 (PMC7570828; doi:10.3390/molecules25184224)
Supplement: Supplementary file 1 [file molecules-25-04224-s001.pdf]

Supporting Information

Cytospyrone and Cytospomarin: Two New Polyketides Isolated from Mangrove Endophytic Fungus, *Cytospora* sp.

Chengwen Wei, [a] Qin Deng,[b] Mengyu Sun,[a] Jing Xu [a, b,]*

[a] Key Laboratory of Advanced Materials of Tropical Island Resources of Ministry of Education, School of Chemical Engineering and Technology, Hainan University, Haikou 570228, P. R. China.

[b] College of Horticulture and Landscape, Hainan University, Haikou 570228, P. R. China

[*] To whom correspondence should be addressed.

Prof. Dr. Jing Xu, Tel.: ++86–898–6627–9226, Fax: ++86–898–6627–9010, E–mail: *happyjing3@163.com*

Contents

Table S1. Gibbs free energies^a and equilibrium populations^b of low-energy conformers of cytospyrone (**1**) --- 3

Table S2. Cartesian coordinates for the low-energy reoptimized MMFF conformers of cytospyrone (**1**) at B3LYP/6-31G(d,p) level of theory in gas---------------------------- 3

Table S3. Gibbs free energies^a and equilibrium populations^b of low-energy conformers of cytospomarin (**2**) --19

Table S4. Cartesian coordinates for the low-energy reoptimized MMFF conformers of cytospomarin (**2**) at B3LYP/6-31G(d,p) level of theory in gas--------------------------19

Table S5. Gibbs free energiesa and equilibrium populationsb of low-energy conformers of cytospomarin (**2**)-- 26

Table S6. Cartesian coordinates for the low-energy reoptimized MMFF conformers of cytospomarin (**2**) at B3LYP/6-31+G(d,p) level of theory in CH_3OH--------------- 26

Figure S1. ^{1}H-NMR of cytospyrone (**1**) -------------------------------------- 34

Figure S2. ^{13}C-NMR of cytospyrone (**1**) ------------------------------------35

Figure S3. DEPT of cytospyrone (**1**) ---35

Figure S4. ^{1}H-^{1}H COSY of cytospyrone (**1**) ------------------------------------36

Figure S5. HMQC of cytospyrone (**1**) --36

Figure S6. HMBC of cytospyrone (**1**) ---37

Figure S7. ROESY of cytospyrone (**1**) --37

Figure S8. HR-ESI-MS of cytospyrone (**1**) -------------------------------------38

Figure S9 CD spectrum of cytospyrone (**1**) -------------------------------------38

Figure S10. ^{1}H-NMR of cytospomarin (**2**) ---------------------------------------38

Figure S11. ^{13}C-NMR of cytospomarin (**2**) --------------------------------------39

Figure S12. DEPT of cytospomarin (**2**) ---39

Figure S13. ^{1}H-^{1}H COSY of cytospomarin (**2**) ---------------------------------40

Figure S14. HMQC of cytospomarin (**2**) ---------------------------------------40

Figure S15. HMBC of cytospomarin (**2**) --41

Figure S16. ROESY of cytospomarin (**2**) ---------------------------------------41

Figure S17. HR-ESI-MS of cytospomarin (**2**) -----------------------------------42

Figure S18. CD spectrum of cytospomarin (**2**)---42

Table S1. Gibbs free energies[a] and equilibrium populations[b] of low-energy conformers of cytospyrone (**1**)

Conformers	In MeOH	
	G[a]	*P* (%)[b]
**1-1**	-725339.39572185	1.63
**1-2**	-725340.33886938	8.03
**1-3**	-725341.33221771	43.01
**1-4**	-725341.3378653	43.42
**1-5**	-725338.49650002	0.36
**1-6**	-725338.01017977	0.16
**1-7**	-725339.77411038	3.09
**1-8**	-725336.54117886	0.01
**1-9**	-725334.89835768	0.29
**1-10**	-725334.89522013	0
**1-11**	-725338.37162553	0.29

[a]B3LYP/6-31G(d,p), in kcal/mol. [b]From *G* values at 298.15K.

Table S2. Cartesian coordinates for the low-energy reoptimized MMFF conformers of cytospyrone (**1**) at B3LYP/6-31G(d,p) level of theory in gas

Conformer **1-1**

**1-1**		Standard Orientation (Ångstroms)			
Center number	Atomic number	Atomic Type	X	Y	Z
1.	8.	0.	-0.895087	-0.272528	0.567277
2.	6.	0.	-1.732054	0.776064	0.675706
3.	6.	0.	-3.049197	0.664222	0.375178
4.	6.	0.	-3.550068	-0.600520	-0.081907
5.	6.	0.	-2.711087	-1.688177	-0.203401
6.	6.	0.	-1.322783	-1.547059	0.140821
7.	6.	0.	-1.035262	2.041814	1.133890
8.	6.	0.	-0.432246	2.878388	-0.049307
9.	6.	0.	0.664974	2.114328	-0.790418
10.	6.	0.	1.900954	2.006583	-0.272899
11.	6.	0.	3.083796	1.219207	-0.802134
12.	6.	0.	3.218200	-0.044372	0.027325
13.	6.	0.	3.124740	-1.321390	-0.374289
14.	6.	0.	3.240357	-2.494833	0.599404
15.	6.	0.	3.297735	-2.135813	2.086083
16.	8.	0.	2.213129	-3.452321	0.355676
17.	6.	0.	2.895396	-1.758505	-1.802162
18.	6.	0.	4.371367	2.066102	-0.731066

19.	6.	0.	0.233095	1.500096	-2.100576
20.	6.	0.	0.006539	4.252317	0.483808
21.	8.	0.	-0.081637	1.754807	2.134259
22.	8.	0.	-0.476391	-2.421816	0.097782
23.	6.	0.	-3.158358	-3.047720	-0.663054
24.	8.	0.	-4.851425	-0.782402	-0.405702
25.	6.	0.	-5.768936	0.302544	-0.293583
26.	1.	0.	-3.690222	1.526846	0.490427
27.	1.	0.	-1.794348	2.669254	1.614652
28.	1.	0.	-1.258112	3.042608	-0.754601
29.	1.	0.	2.103118	2.513467	0.671006
30.	1.	0.	2.912152	0.956693	-1.847907
31.	1.	0.	3.391208	0.152375	1.084880
32.	1.	0.	4.169598	-3.028905	0.346849
33.	1.	0.	2.399904	-1.588700	2.394580
34.	1.	0.	4.175169	-1.529723	2.334705
35.	1.	0.	3.344808	-3.061219	2.665604
36.	1.	0.	1.351939	-3.001857	0.413052
37.	1.	0.	1.903597	-2.208176	-1.909727
38.	1.	0.	3.614904	-2.537728	-2.080587
39.	1.	0.	2.990205	-0.941906	-2.520617
40.	1.	0.	5.225563	1.505124	-1.122055
41.	1.	0.	4.267607	2.989828	-1.310255
42.	1.	0.	4.600899	2.344064	0.304309
43.	1.	0.	-0.050993	2.285694	-2.813751
44.	1.	0.	1.006203	0.887370	-2.564790
45.	1.	0.	-0.652703	0.867766	-1.968865
46.	1.	0.	-0.854511	4.808299	0.871515
47.	1.	0.	0.725283	4.150876	1.299937
48.	1.	0.	0.466649	4.845152	-0.311188
49.	1.	0.	0.571504	1.160388	1.731984
50.	1.	0.	-2.590656	-3.361569	-1.545275
51.	1.	0.	-2.967079	-3.797631	0.111796
52.	1.	0.	-4.220936	-3.050672	-0.904675
53.	1.	0.	-6.733343	-0.096759	-0.607996
54.	1.	0.	-5.492179	1.134171	-0.951383
55.	1.	0.	-5.846899	0.659299	0.739527

Conformer **1-2**

**1-2**		Standard Orientation (Ångstroms)			
Center number	Atom number	Type	X	Y	Z
1.	8.	0.	0.942076	-0.216214	-0.699264
2.	6.	0.	1.799227	0.819418	-0.645090
3.	6.	0.	3.086890	0.653150	-0.254652
4.	6.	0.	3.531794	-0.657787	0.122245
5.	6.	0.	2.671493	-1.734755	0.075624
6.	6.	0.	1.319415	-1.536126	-0.369129
7.	6.	0.	1.159220	2.136892	-1.035910
8.	6.	0.	0.494237	2.881840	0.173798
9.	6.	0.	-0.665556	2.086037	0.772080
10.	6.	0.	-1.875551	2.070308	0.186669
11.	6.	0.	-3.107170	1.290749	0.613665
12.	6.	0.	-3.128724	-0.042284	-0.110929
13.	6.	0.	-3.236696	-1.274102	0.409325
14.	6.	0.	-3.203158	-2.536323	-0.455915
15.	6.	0.	-3.052029	-2.319005	-1.962117
16.	8.	0.	-2.205088	-3.438228	0.022114
17.	6.	0.	-3.399033	-1.577874	1.879492
18.	6.	0.	-4.384266	2.101761	0.311025
19.	6.	0.	-0.333940	1.329907	2.036687
20.	6.	0.	0.119835	4.307632	-0.263117
21.	8.	0.	0.269932	1.962874	-2.118172
22.	8.	0.	0.462987	-2.392707	-0.490431
23.	6.	0.	3.063713	-3.138455	0.444042
24.	8.	0.	4.800196	-0.895846	0.529502
25.	6.	0.	5.731848	0.179601	0.614396
26.	1.	0.	3.747139	1.508765	-0.240255
27.	1.	0.	1.960831	2.784758	-1.408476
28.	1.	0.	1.273568	2.961745	0.943742
29.	1.	0.	-2.014046	2.661868	-0.718280
30.	1.	0.	-3.069910	1.120625	1.692522
31.	1.	0.	-3.035663	0.053431	-1.192035
32.	1.	0.	-4.151090	-3.067692	-0.282303
33.	1.	0.	-2.104026	-1.821220	-2.192131
34.	1.	0.	-3.870945	-1.722873	-2.377560
35.	1.	0.	-3.047991	-3.293150	-2.457832
36.	1.	0.	-1.337116	-3.034168	-0.154373
37.	1.	0.	-2.627597	-2.284040	2.199395
38.	1.	0.	-4.363203	-2.071833	2.060055

39.	1.	0.	-3.359244	-0.691581	2.515656
40.	1.	0.	-5.274321	1.562481	0.647885
41.	1.	0.	-4.359558	3.077036	0.809499
42.	1.	0.	-4.489407	2.278063	-0.765914
43.	1.	0.	-0.115240	2.029365	2.855087
44.	1.	0.	-1.135730	0.666272	2.360534
45.	1.	0.	0.565551	0.717572	1.905638
46.	1.	0.	1.015792	4.871443	-0.546403
47.	1.	0.	-0.546833	4.295507	-1.128119
48.	1.	0.	-0.378043	4.842133	0.550227
49.	1.	0.	-0.418755	1.351021	-1.813960
50.	1.	0.	2.350037	-3.557649	1.159899
51.	1.	0.	3.042998	-3.791674	-0.435520
52.	1.	0.	4.063935	-3.165797	0.875992
53.	1.	0.	6.660209	-0.265637	0.972560
54.	1.	0.	5.401006	0.944171	1.326317
55.	1.	0.	5.907546	0.638545	-0.364968

Conformer **1-3**

**1-3**		Standard Orientation (Ångstroms)			
Center number	Atom number	Type	X	Y	Z
1.	8.	0.	0.995833	-0.244008	-0.741134
2.	6.	0.	1.932174	0.717078	-0.635315
3.	6.	0.	3.189508	0.434752	-0.213664
4.	6.	0.	3.512132	-0.916694	0.144546
5.	6.	0.	2.565987	-1.915945	0.051413
6.	6.	0.	1.249476	-1.592399	-0.423859
7.	6.	0.	1.410720	2.092022	-1.003136
8.	6.	0.	0.764927	2.849185	0.210189
9.	6.	0.	-0.461530	2.118990	0.755305
10.	6.	0.	-1.655868	2.200389	0.144682
11.	6.	0.	-2.939406	1.481925	0.531411
12.	6.	0.	-2.940169	0.090987	-0.069827
13.	6.	0.	-3.199200	-1.080915	0.526110
14.	6.	0.	-3.113550	-2.378759	-0.285157
15.	6.	0.	-4.501809	-2.908612	-0.660828
16.	8.	0.	-2.393640	-2.238064	-1.495049
17.	6.	0.	-3.591258	-1.257909	1.972269
18.	6.	0.	-4.166125	2.288723	0.058027
19.	6.	0.	-0.219180	1.305544	2.004698

20.	6.	0.	0.501562	4.308372	-0.196569
21.	8.	0.	0.548360	2.022539	-2.117501
22.	8.	0.	0.325664	-2.373613	-0.579413
23.	6.	0.	2.826180	-3.355100	0.398864
24.	8.	0.	4.743575	-1.268683	0.581139
25.	6.	0.	5.767094	-0.282326	0.687984
26.	1.	0.	3.917148	1.232085	-0.156829
27.	1.	0.	2.272976	2.685340	-1.328070
28.	1.	0.	1.526499	2.854591	1.001788
29.	1.	0.	-1.737079	2.825328	-0.744437
30.	1.	0.	-2.992240	1.411038	1.622771
31.	1.	0.	-2.682840	0.042600	-1.127245
32.	1.	0.	-2.622827	-3.130451	0.359081
33.	1.	0.	-4.999584	-2.192227	-1.321481
34.	1.	0.	-5.130205	-3.064290	0.221292
35.	1.	0.	-4.404507	-3.859658	-1.191846
36.	1.	0.	-1.450715	-2.158212	-1.261047
37.	1.	0.	-4.605005	-1.668269	2.062357
38.	1.	0.	-3.570434	-0.323645	2.537963
39.	1.	0.	-2.923872	-1.971521	2.472909
40.	1.	0.	-5.094072	1.799870	0.367862
41.	1.	0.	-4.153917	3.304168	0.469893
42.	1.	0.	-4.181588	2.368610	-1.034998
43.	1.	0.	-0.017000	1.965995	2.859189
44.	1.	0.	0.663890	0.665737	1.894765
45.	1.	0.	-1.060295	0.661309	2.261114
46.	1.	0.	1.441707	4.816334	-0.439475
47.	1.	0.	-0.138868	4.365466	-1.079379
48.	1.	0.	0.018211	4.854118	0.618065
49.	1.	0.	-0.201704	1.465298	-1.855971
50.	1.	0.	2.679932	-3.995552	-0.477509
51.	1.	0.	3.840928	-3.491793	0.771780
52.	1.	0.	2.117152	-3.700800	1.158259
53.	1.	0.	5.498207	0.501447	1.405057
54.	1.	0.	5.994406	0.168816	-0.284261
55.	1.	0.	6.647366	-0.812933	1.051211

Conformer **1-4**

**1 -4**	Standard Orientation (Ångstroms)

Center number	Atom number	Type	X	Y	Z
1.	8.	0.	0.995791	-0.243995	-0.740791
2.	6.	0.	1.932229	0.717049	-0.635331
3.	6.	0.	3.189636	0.434687	-0.213939
4.	6.	0.	3.512241	-0.916747	0.144339
5.	6.	0.	2.565932	-1.915886	0.051763
6.	6.	0.	1.249432	-1.592334	-0.423583
7.	6.	0.	1.410761	2.091942	-1.003292
8.	6.	0.	0.764976	2.849244	0.209974
9.	6.	0.	-0.461372	2.118977	0.755216
10.	6.	0.	-1.655835	2.200383	0.144835
11.	6.	0.	-2.939225	1.481810	0.531821
12.	6.	0.	-2.940173	0.090979	-0.069700
13.	6.	0.	-3.199255	-1.081003	0.526070
14.	6.	0.	-3.114015	-2.378699	-0.285486
15.	6.	0.	-4.502466	-2.907674	-0.661690
16.	8.	0.	-2.393715	-2.238099	-1.495138
17.	6.	0.	-3.590923	-1.258239	1.972297
18.	6.	0.	-4.166130	2.288674	0.059047
19.	6.	0.	-0.218844	1.305453	2.004547
20.	6.	0.	0.501512	4.308354	-0.196973
21.	8.	0.	0.548323	2.022253	-2.117575
22.	8.	0.	0.325593	-2.373551	-0.579053
23.	6.	0.	2.826131	-3.354997	0.399386
24.	8.	0.	4.743837	-1.268862	0.580388
25.	6.	0.	5.767142	-0.282375	0.688063
26.	1.	0.	3.917399	1.231942	-0.157567
27.	1.	0.	2.272979	2.685254	-1.328346
28.	1.	0.	1.526578	2.854812	1.001541
29.	1.	0.	-1.737302	2.825422	-0.744191
30.	1.	0.	-2.991666	1.410670	1.623178
31.	1.	0.	-2.682991	0.042767	-1.127163
32.	1.	0.	-2.623900	-3.130835	0.358705
33.	1.	0.	-4.999616	-2.190860	-1.322346
34.	1.	0.	-5.131220	-3.063158	0.220210
35.	1.	0.	-4.405577	-3.858677	-1.192863
36.	1.	0.	-1.450862	-2.158172	-1.260866
37.	1.	0.	-4.604316	-1.669420	2.062572
38.	1.	0.	-3.570735	-0.323938	2.537949
39.	1.	0.	-2.922881	-1.971312	2.472838
40.	1.	0.	-5.093950	1.799710	0.369089
41.	1.	0.	-4.153812	3.304021	0.471149

42.	1.	0.	-4.181981	2.368819	-1.033954
43.	1.	0.	-0.018530	1.965919	2.859472
44.	1.	0.	0.665371	0.667150	1.895192
45.	1.	0.	-1.059163	0.659775	2.259957
46.	1.	0.	1.441629	4.816326	-0.439962
47.	1.	0.	-0.138924	4.365312	-1.079785
48.	1.	0.	0.018154	4.854185	0.617601
49.	1.	0.	-0.201883	1.465328	-1.855766
50.	1.	0.	2.689586	-3.994709	-0.479163
51.	1.	0.	3.837766	-3.490046	0.781274
52.	1.	0.	2.110727	-3.703118	1.151565
53.	1.	0.	5.497839	0.501053	1.405361
54.	1.	0.	5.994772	0.169217	-0.283898
55.	1.	0.	6.647371	-0.812988	1.051386

Conformer **1-5**

**1-5**		Standard Orientation (Ångstroms)			
Center number	Atom number	Type	X	Y	Z
1.	8.	0.	0.873459	0.208998	-0.972157
2.	6.	0.	1.898070	-0.621750	-0.703009
3.	6.	0.	3.037821	-0.184821	-0.112910
4.	6.	0.	3.141960	1.200211	0.243167
5.	6.	0.	2.108829	2.073788	-0.019098
6.	6.	0.	0.911614	1.579694	-0.644549
7.	6.	0.	1.644109	-2.045952	-1.148956
8.	6.	0.	0.747689	-2.875329	-0.177224
9.	6.	0.	-0.707557	-2.415629	-0.196406
10.	6.	0.	-1.337097	-1.988253	0.908713
11.	6.	0.	-2.780077	-1.532004	1.030895
12.	6.	0.	-2.865939	-0.026691	0.867575
13.	6.	0.	-3.566705	0.671872	-0.037774
14.	6.	0.	-3.518366	2.198924	-0.114489
15.	6.	0.	-2.861399	2.920134	1.063293
16.	8.	0.	-2.929017	2.602286	-1.352636
17.	6.	0.	-4.443901	0.073378	-1.111226
18.	6.	0.	-3.361404	-1.962823	2.393700
19.	6.	0.	-1.383047	-2.496034	-1.548996
20.	6.	0.	1.391997	-3.006541	1.206674
21.	8.	0.	2.871778	-2.757539	-1.274596

22.	8.	0.	-0.072844	2.238782	-0.929021
23.	6.	0.	2.144092	3.539117	0.313962
24.	8.	0.	4.245911	1.705044	0.842045
25.	6.	0.	5.345172	0.846552	1.137125
26.	1.	0.	3.823932	-0.900512	0.074885
27.	1.	0.	1.130645	-1.989291	-2.118414
28.	1.	0.	0.764034	-3.874542	-0.638091
29.	1.	0.	-0.772792	-1.930294	1.837749
30.	1.	0.	-3.375210	-2.012825	0.250205
31.	1.	0.	-2.268029	0.523942	1.592652
32.	1.	0.	-4.558876	2.548638	-0.183866
33.	1.	0.	-1.799920	2.666357	1.139681
34.	1.	0.	-3.350994	2.678164	2.012379
35.	1.	0.	-2.932055	3.998758	0.899287
36.	1.	0.	-1.988532	2.354071	-1.311286
37.	1.	0.	-4.377674	-1.013633	-1.181668
38.	1.	0.	-4.173830	0.505713	-2.079670
39.	1.	0.	-5.496678	0.331604	-0.933181
40.	1.	0.	-4.412420	-1.670758	2.477285
41.	1.	0.	-3.291020	-3.047972	2.526831
42.	1.	0.	-2.817308	-1.488095	3.218883
43.	1.	0.	-2.459723	-2.656399	-1.464428
44.	1.	0.	-1.243670	-1.568049	-2.116855
45.	1.	0.	-0.974187	-3.316581	-2.150138
46.	1.	0.	2.405274	-3.402877	1.109914
47.	1.	0.	1.451330	-2.046661	1.728570
48.	1.	0.	0.813612	-3.693564	1.830150
49.	1.	0.	3.376372	-2.354828	-1.994046
50.	1.	0.	1.419322	3.778032	1.100697
51.	1.	0.	3.135641	3.840974	0.651181
52.	1.	0.	1.861978	4.134427	-0.559612
53.	1.	0.	5.771180	0.409427	0.227076
54.	1.	0.	5.057783	0.047219	1.828970
55.	1.	0.	6.092355	1.481949	1.612982

Conformer **1-6**

**1-6**		Standard Orientation (Ångstroms)			
Center number	Atom number	Type	X	Y	Z
1.	8.	0.	0.890952	0.172725	-0.878683
2.	6.	0.	1.933787	-0.651350	-0.661893

3.	6.	0.	3.097666	-0.212093	-0.127009
4.	6.	0.	3.212926	1.174250	0.218590
5.	6.	0.	2.162275	2.042542	0.011988
6.	6.	0.	0.939903	1.543372	-0.555973
7.	6.	0.	1.654199	-2.068441	-1.094635
8.	6.	0.	0.702869	-2.858048	-0.130390
9.	6.	0.	-0.747991	-2.390362	-0.202023
10.	6.	0.	-1.419229	-1.980502	0.885130
11.	6.	0.	-2.863340	-1.518315	0.960564
12.	6.	0.	-2.931331	-0.009103	0.830064
13.	6.	0.	-3.594383	0.715899	-0.082615
14.	6.	0.	-3.523137	2.243367	-0.126336
15.	6.	0.	-2.900629	2.931145	1.089473
16.	8.	0.	-2.880396	2.660377	-1.332868
17.	6.	0.	-4.440151	0.150416	-1.198141
18.	6.	0.	-3.499738	-1.977715	2.289026
19.	6.	0.	-1.368431	-2.445985	-1.581154
20.	6.	0.	1.305093	-2.964041	1.274131
21.	8.	0.	2.919444	-2.714549	-1.201556
22.	8.	0.	-0.059858	2.198715	-0.794507
23.	6.	0.	2.207468	3.508650	0.340663
24.	8.	0.	4.344778	1.685515	0.756433
25.	6.	0.	5.472545	0.838218	0.965088
26.	1.	0.	3.901301	-0.920092	0.000910
27.	1.	0.	1.173887	-2.009470	-2.081425
28.	1.	0.	0.702238	-3.875159	-0.563342
29.	1.	0.	-0.891570	-1.940108	1.836230
30.	1.	0.	-3.429963	-1.976141	0.145593
31.	1.	0.	-2.351945	0.519532	1.585654
32.	1.	0.	-4.555166	2.609811	-0.228419
33.	1.	0.	-1.846127	2.660982	1.198438
34.	1.	0.	-3.428137	2.676635	2.014734
35.	1.	0.	-2.951285	4.013787	0.945694
36.	1.	0.	-1.947458	2.391201	-1.260896
37.	1.	0.	-4.129191	0.597322	-2.147546
38.	1.	0.	-5.495078	0.420107	-1.053098
39.	1.	0.	-4.386411	-0.935992	-1.287393
40.	1.	0.	-4.551431	-1.680531	2.338313
41.	1.	0.	-3.441197	-3.066195	2.399327
42.	1.	0.	-2.985705	-1.525833	3.145673
43.	1.	0.	-1.172281	-1.522292	-2.138826
44.	1.	0.	-0.963925	-3.278649	-2.169407
45.	1.	0.	-2.452242	-2.569500	-1.541797

46.	1.	0.	2.331162	-3.335236	1.214382
47.	1.	0.	1.331061	-1.994383	1.779628
48.	1.	0.	0.724276	-3.653225	1.892544
49.	1.	0.	2.753988	-3.645950	-1.396686
50.	1.	0.	1.906228	4.103227	-0.527259
51.	1.	0.	1.501345	3.750195	1.143265
52.	1.	0.	3.207297	3.809662	0.653175
53.	1.	0.	6.244937	1.480066	1.389396
54.	1.	0.	5.834596	0.410386	0.023844
55.	1.	0.	5.245221	0.032163	1.671466

Conformer **1-7**

**1-7**		Standard Orientation (Ångstroms)			
Center number	Atom number	Type	X	Y	Z
1.	8.	0.	1.042786	0.099797	-1.332838
2.	6.	0.	2.134781	-0.393129	-0.721825
3.	6.	0.	2.936460	0.386535	0.044481
4.	6.	0.	2.572908	1.760165	0.241474
5.	6.	0.	1.434919	2.279764	-0.340098
6.	6.	0.	0.623088	1.432258	-1.169043
7.	6.	0.	2.329728	-1.870707	-0.982233
8.	6.	0.	1.511201	-2.782359	-0.011709
9.	6.	0.	0.005062	-2.571801	-0.166812
10.	6.	0.	-0.746856	-2.088205	0.833684
11.	6.	0.	-2.240563	-1.830420	0.848339
12.	6.	0.	-2.527175	-0.359493	0.606352
13.	6.	0.	-3.504368	0.177733	-0.136845
14.	6.	0.	-3.652205	1.699582	-0.233291
15.	6.	0.	-4.728797	2.225713	0.721446
16.	8.	0.	-2.462852	2.407427	0.073681
17.	6.	0.	-4.533109	-0.602735	-0.917110
18.	6.	0.	-2.836151	-2.282884	2.201051
19.	6.	0.	-0.554511	-2.936679	-1.524760
20.	6.	0.	2.049983	-2.699543	1.421050
21.	8.	0.	3.697462	-2.239201	-0.842636
22.	8.	0.	-0.398270	1.746498	-1.760106
23.	6.	0.	0.967910	3.697650	-0.159187
24.	8.	0.	3.316392	2.594283	1.005048
25.	6.	0.	4.485047	2.103201	1.656957
26.	1.	0.	3.806977	-0.062708	0.498459

27.	1.	0.	1.977207	-2.063379	-2.004739
28.	1.	0.	1.730656	-3.795529	-0.380256
29.	1.	0.	-0.252046	-1.821650	1.766189
30.	1.	0.	-2.716647	-2.424002	0.062220
31.	1.	0.	-1.862450	0.338613	1.113282
32.	1.	0.	-3.970006	1.931171	-1.266116
33.	1.	0.	-4.423609	2.036890	1.755103
34.	1.	0.	-5.694062	1.740080	0.549436
35.	1.	0.	-4.852577	3.304175	0.586835
36.	1.	0.	-1.812205	2.207262	-0.624311
37.	1.	0.	-4.468466	-0.366922	-1.987485
38.	1.	0.	-5.551925	-0.341429	-0.604050
39.	1.	0.	-4.426370	-1.683421	-0.804117
40.	1.	0.	-3.920781	-2.140660	2.213189
41.	1.	0.	-2.620517	-3.339947	2.393035
42.	1.	0.	-2.415569	-1.697215	3.026540
43.	1.	0.	-0.425438	-2.113208	-2.237345
44.	1.	0.	-0.046188	-3.815335	-1.939829
45.	1.	0.	-1.622647	-3.159517	-1.487142
46.	1.	0.	3.111520	-2.954210	1.435694
47.	1.	0.	1.936487	-1.699035	1.848742
48.	1.	0.	1.516343	-3.403504	2.065116
49.	1.	0.	4.197641	-1.793020	-1.539088
50.	1.	0.	-0.017808	3.718067	0.318783
51.	1.	0.	1.668958	4.266230	0.451486
52.	1.	0.	0.857323	4.192159	-1.129900
53.	1.	0.	4.245379	1.298980	2.361265
54.	1.	0.	4.891648	2.952497	2.206352
55.	1.	0.	5.230927	1.750253	0.935937

Conformer **1-8**

**1-8**		Standard Orientation (Ångstroms)			
Center number	Atom number	Type	X	Y	Z
1.	8.	0.	-2.700215	1.164233	-0.417989
2.	6.	0.	-2.229748	0.437573	0.613717
3.	6.	0.	-1.096626	0.786961	1.266040
4.	6.	0.	-0.356654	1.914837	0.786365
5.	6.	0.	-0.791353	2.668116	-0.277252
6.	6.	0.	-2.034266	2.320429	-0.927571
7.	6.	0.	-3.059358	-0.794212	0.889515

8.	6.	0.	-2.694869	-2.010154	-0.039839
9.	6.	0.	-1.247464	-2.456084	0.169850
10.	6.	0.	-0.260908	-2.027624	-0.632270
11.	6.	0.	1.221776	-2.326322	-0.581476
12.	6.	0.	2.008157	-1.029713	-0.557930
13.	6.	0.	3.128917	-0.748731	0.123583
14.	6.	0.	3.825015	0.603400	-0.062695
15.	6.	0.	5.064775	0.486974	-0.954554
16.	8.	0.	3.001757	1.595408	-0.659476
17.	6.	0.	3.835404	-1.700486	1.059544
18.	6.	0.	1.628660	-3.168046	-1.817473
19.	6.	0.	-1.046729	-3.380180	1.349336
20.	6.	0.	-3.102512	-1.771723	-1.500795
21.	8.	0.	-4.446040	-0.500511	0.838926
22.	8.	0.	-2.577971	2.882568	-1.849885
23.	6.	0.	-0.017411	3.842043	-0.813634
24.	8.	0.	0.836536	2.276550	1.342861
25.	6.	0.	1.257709	1.651552	2.560170
26.	1.	0.	-0.746154	0.176217	2.084416
27.	1.	0.	-2.853779	-1.095425	1.921887
28.	1.	0.	-3.342214	-2.811624	0.341611
29.	1.	0.	-0.532095	-1.373004	-1.460348
30.	1.	0.	1.454494	-2.916056	0.309762
31.	1.	0.	1.637317	-0.248165	-1.220159
32.	1.	0.	4.158109	0.943454	0.935746
33.	1.	0.	4.764945	0.166460	-1.956567
34.	1.	0.	5.781133	-0.236651	-0.555379
35.	1.	0.	5.558313	1.459457	-1.033323
36.	1.	0.	2.296176	1.823434	-0.034302
37.	1.	0.	3.231746	-2.576337	1.306898
38.	1.	0.	4.106221	-1.201525	1.999046
39.	1.	0.	4.773581	-2.066340	0.623571
40.	1.	0.	2.698342	-3.398023	-1.794765
41.	1.	0.	1.067250	-4.107435	-1.848844
42.	1.	0.	1.423685	-2.621359	-2.744255
43.	1.	0.	-1.482520	-2.961551	2.265964
44.	1.	0.	-1.557962	-4.336615	1.179317
45.	1.	0.	0.003057	-3.597857	1.553790
46.	1.	0.	-4.175677	-1.570446	-1.563097
47.	1.	0.	-2.581954	-0.928868	-1.961151
48.	1.	0.	-2.895271	-2.664054	-2.097950
49.	1.	0.	-4.593989	0.051863	0.057628
50.	1.	0.	-0.644262	4.406977	-1.504214

51.	1.	0.	0.313946	4.498947	-0.003969
52.	1.	0.	0.879209	3.514271	-1.353579
53.	1.	0.	0.502547	1.766519	3.344034
54.	1.	0.	2.167507	2.174602	2.855695
55.	1.	0.	1.480670	0.591495	2.401269

Conformer **1-9**

**1-9**		Standard Orientation (Ångstroms)			
Center number	Atom number	Type	X	Y	Z
1.	8.	0.	-0.968130	0.349736	-1.330655
2.	6.	0.	-2.091358	0.245984	-0.598493
3.	6.	0.	-2.485991	1.239711	0.232856
4.	6.	0.	-1.662773	2.409919	0.346900
5.	6.	0.	-0.494244	2.528791	-0.377682
6.	6.	0.	-0.111743	1.469030	-1.266716
7.	6.	0.	-2.779540	-1.087875	-0.790323
8.	6.	0.	-2.324519	-2.133608	0.285739
9.	6.	0.	-0.820019	-2.399883	0.310991
10.	6.	0.	-0.081898	-1.831842	1.280223
11.	6.	0.	1.416115	-1.919136	1.573024
12.	6.	0.	2.255566	-1.196678	0.535347
13.	6.	0.	3.009182	-0.098818	0.701331
14.	6.	0.	3.846563	0.502105	-0.431212
15.	6.	0.	3.947547	-0.325999	-1.712386
16.	8.	0.	3.411611	1.833751	-0.730197
17.	6.	0.	3.141326	0.675939	1.991149
18.	6.	0.	1.921069	-3.365560	1.784855
19.	6.	0.	-0.276576	-3.323813	-0.758197
20.	6.	0.	-3.163473	-3.413483	0.130090
21.	8.	0.	-2.640990	-1.546271	-2.125168
22.	8.	0.	0.871549	1.423543	-1.987907
23.	6.	0.	0.420246	3.720790	-0.305011
24.	8.	0.	-1.992924	3.440124	1.158824
25.	6.	0.	-3.180934	3.374763	1.943398
26.	1.	0.	-3.403154	1.125967	0.793351
27.	1.	0.	-3.854084	-0.930131	-0.632194
28.	1.	0.	-2.588020	-1.674181	1.247384
29.	1.	0.	-0.621672	-1.193052	1.980020
30.	1.	0.	1.554721	-1.411742	2.532213
31.	1.	0.	2.243927	-1.658739	-0.448783

32.	1.	0.	4.862928	0.634159	-0.031565
33.	1.	0.	2.966825	-0.435916	-2.186256
34.	1.	0.	4.359982	-1.321957	-1.522284
35.	1.	0.	4.602776	0.192533	-2.417370
36.	1.	0.	2.580489	1.753244	-1.234052
37.	1.	0.	3.036624	1.745379	1.784991
38.	1.	0.	4.140655	0.535814	2.425488
39.	1.	0.	2.406850	0.395884	2.748725
40.	1.	0.	2.954565	-3.351941	2.145486
41.	1.	0.	1.304388	-3.887134	2.524289
42.	1.	0.	1.900995	-3.950563	0.861810
43.	1.	0.	-0.744808	-3.124698	-1.725544
44.	1.	0.	0.802408	-3.234377	-0.878512
45.	1.	0.	-0.498173	-4.372307	-0.521187
46.	1.	0.	-4.229999	-3.192730	0.248853
47.	1.	0.	-3.028094	-3.860983	-0.857083
48.	1.	0.	-2.885303	-4.150939	0.888898
49.	1.	0.	-1.765063	-1.273482	-2.435592
50.	1.	0.	1.436255	3.408847	-0.042663
51.	1.	0.	0.484705	4.217569	-1.279542
52.	1.	0.	0.065222	4.440665	0.431976
53.	1.	0.	-3.215085	4.311331	2.500150
54.	1.	0.	-3.151825	2.537292	2.649539
55.	1.	0.	-4.075239	3.297101	1.314989

Conformer **1-10**

**1-10**		Standard Orientation (Ångstroms)			
Center number	Atom number	Type	X	Y	Z
1.	8.	0.	-0.967796	0.351086	-1.331084
2.	6.	0.	-2.090914	0.248701	-0.598589
3.	6.	0.	-2.483887	1.242779	0.233135
4.	6.	0.	-1.658989	2.411791	0.347169
5.	6.	0.	-0.490581	2.529247	-0.377868
6.	6.	0.	-0.109779	1.469073	-1.267106
7.	6.	0.	-2.780958	-1.084199	-0.790486
8.	6.	0.	-2.327479	-2.130510	0.285679
9.	6.	0.	-0.823323	-2.398723	0.311072
10.	6.	0.	-0.084586	-1.831638	1.280398
11.	6.	0.	1.413295	-1.920761	1.573294
12.	6.	0.	2.253619	-1.199489	0.535481

13.	6.	0.	3.008769	-0.102659	0.701268
14.	6.	0.	3.846627	0.497121	-0.431539
15.	6.	0.	3.945834	-0.330999	-1.712824
16.	8.	0.	3.413464	1.829453	-0.730199
17.	6.	0.	3.142390	0.671881	1.991057
18.	6.	0.	1.916525	-3.367747	1.785435
19.	6.	0.	-0.280942	-3.323287	-0.758100
20.	6.	0.	-3.168124	-3.409269	0.129953
21.	8.	0.	-2.642899	-1.542895	-2.125250
22.	8.	0.	0.873344	1.422273	-1.988472
23.	6.	0.	0.425531	3.720009	-0.305371
24.	8.	0.	-1.987327	3.442258	1.159524
25.	6.	0.	-3.175382	3.378625	1.944197
26.	1.	0.	-3.400961	1.130140	0.794003
27.	1.	0.	-3.855293	-0.924923	-0.632447
28.	1.	0.	-2.590464	-1.670703	1.247281
29.	1.	0.	-0.623630	-1.192197	1.980162
30.	1.	0.	1.552499	-1.413358	2.532394
31.	1.	0.	2.241140	-1.661566	-0.448634
32.	1.	0.	4.863358	0.627621	-0.032311
33.	1.	0.	2.964745	-0.439415	-2.186278
34.	1.	0.	4.356893	-1.327576	-1.522981
35.	1.	0.	4.601541	0.186618	-2.418037
36.	1.	0.	2.582358	1.750154	-1.234283
37.	1.	0.	3.038514	1.741430	1.785062
38.	1.	0.	4.141876	0.530838	2.424736
39.	1.	0.	2.408159	0.392390	2.749078
40.	1.	0.	2.949971	-3.355245	2.146245
41.	1.	0.	1.299112	-3.888500	2.524837
42.	1.	0.	1.895967	-3.952889	0.862487
43.	1.	0.	-0.748337	-3.123069	-1.725627
44.	1.	0.	0.798255	-3.235692	-0.877827
45.	1.	0.	-0.504463	-4.371471	-0.521528
46.	1.	0.	-4.234356	-3.187093	0.248705
47.	1.	0.	-3.033309	-3.856887	-0.857241
48.	1.	0.	-2.890946	-4.147131	0.888730
49.	1.	0.	-1.766814	-1.270828	-2.435852
50.	1.	0.	1.440913	3.406873	-0.041976
51.	1.	0.	0.491484	4.215803	-1.280297
52.	1.	0.	0.070923	4.440978	0.430731
53.	1.	0.	-3.208097	4.315262	2.500920
54.	1.	0.	-3.147368	2.541152	2.650379
55.	1.	0.	-4.069847	3.302215	1.315883

Conformer **1-11**

**1-11**		Standard Orientation (Ångstroms)			
Center number	Atom number	Type	X	Y	Z
1.	8.	0.	0.920360	-0.405403	-0.468276
2.	6.	0.	1.722509	0.648576	-0.707230
3.	6.	0.	3.056774	0.588188	-0.474413
4.	6.	0.	3.612695	-0.622722	0.057629
5.	6.	0.	2.808565	-1.712721	0.318018
6.	6.	0.	1.401832	-1.628706	0.039290
7.	6.	0.	0.967892	1.862985	-1.210154
8.	6.	0.	0.470240	2.800840	-0.052962
9.	6.	0.	-0.548530	2.107296	0.849384
10.	6.	0.	-1.839238	2.000783	0.490705
11.	6.	0.	-2.949858	1.298299	1.257390
12.	6.	0.	-2.940973	-0.220004	1.065370
13.	6.	0.	-3.089250	-0.936735	-0.058705
14.	6.	0.	-3.036350	-2.462584	0.013744
15.	6.	0.	-4.361676	-3.121966	-0.373484
16.	8.	0.	-2.050371	-2.986519	-0.866830
17.	6.	0.	-3.286827	-0.374232	-1.445650
18.	6.	0.	-4.321643	1.930099	0.944763
19.	6.	0.	0.009440	1.554195	2.139594
20.	6.	0.	-0.024440	4.120679	-0.667350
21.	8.	0.	-0.072026	1.487740	-2.085861
22.	8.	0.	0.579485	-2.514535	0.200513
23.	6.	0.	3.313582	-3.018752	0.864768
24.	8.	0.	4.933538	-0.750278	0.322018
25.	6.	0.	5.818883	0.334444	0.054292
26.	1.	0.	3.668744	1.451879	-0.693057
27.	1.	0.	1.671160	2.448404	-1.813688
28.	1.	0.	1.355975	3.025775	0.556801
29.	1.	0.	-2.146108	2.464927	-0.444542
30.	1.	0.	-2.769611	1.466206	2.325614
31.	1.	0.	-2.792774	-0.786155	1.985445
32.	1.	0.	-2.806433	-2.740739	1.054841
33.	1.	0.	-4.615850	-2.901235	-1.414500
34.	1.	0.	-5.176006	-2.772636	0.268813
35.	1.	0.	-4.272114	-4.207582	-0.275602

36.	1.	0.	-1.173314	-2.740163	-0.519777
37.	1.	0.	-3.052526	0.689074	-1.517755
38.	1.	0.	-4.323156	-0.503364	-1.782519
39.	1.	0.	-2.656752	-0.926740	-2.150177
40.	1.	0.	-5.110547	1.450329	1.531453
41.	1.	0.	-4.320274	2.999838	1.182345
42.	1.	0.	-4.581757	1.820183	-0.112276
43.	1.	0.	0.387215	2.369115	2.772063
44.	1.	0.	0.859378	0.887339	1.955558
45.	1.	0.	-0.726658	0.991020	2.714355
46.	1.	0.	0.796378	4.641708	-1.172861
47.	1.	0.	-0.805456	3.942441	-1.409863
48.	1.	0.	-0.424755	4.781190	0.106241
49.	1.	0.	-0.662815	0.899491	-1.589809
50.	1.	0.	3.049644	-3.842076	0.193165
51.	1.	0.	4.395229	-2.997784	0.995470
52.	1.	0.	2.844163	-3.241530	1.829065
53.	1.	0.	5.565332	1.218247	0.650552
54.	1.	0.	5.824872	0.596159	-1.009654
55.	1.	0.	6.809167	-0.019130	0.341795

Table S3. Gibbs free energies[a] and equilibrium populations[b] of low-energy conformers of cytospomarin (**2**)

Conformers	In MeOH	
	G^a	P (%)[b]
**2-1**	-599254.34919792	11.53
**2-2**	-599254.5926718	17.39
**2-3**	-599254.13145195	7.98
**2-4**	-599254.68177822	20.22
**2-5**	-599253.19520703	1.64
**2-6**	-599254.72884147	21.89
**2-7**	-599254.6554228	19.34

[a]B3LYP/6-31G(d,p), in kcal/mol. [b]From *G* values at 298.15K.

Table S4. Cartesian coordinates for the low-energy reoptimized MMFF conformers of cytospomarin (**2**) at B3LYP/6-31G(d,p) level of theory in gas

Conformer **2-1**

**2-1**		Standard Orientation (Ångstroms)			
No.	Atom	Type	X	Y	Z
1.	6.	0.	-3.299084	0.862809	0.197751
2.	6.	0.	-3.165693	-0.505707	-0.039249
3.	6.	0.	-1.906543	-1.090102	-0.271458

4.	6.	0.	-0.762265	-0.296083	-0.311974
5.	6.	0.	-0.886472	1.099096	-0.064602
6.	6.	0.	-2.162893	1.673068	0.200317
7.	6.	0.	0.552173	-0.811693	-0.605633
8.	6.	0.	1.617871	0.012279	-0.617353
9.	8.	0.	1.496522	1.354960	-0.351341
10.	6.	0.	0.281362	1.944947	-0.076026
11.	6.	0.	3.037622	-0.362971	-0.906492
12.	6.	0.	3.970640	-0.201353	0.304045
13.	8.	0.	3.499593	-1.120947	1.290228
14.	6.	0.	5.428422	-0.477193	-0.064162
15.	8.	0.	0.290969	3.155519	0.138284
16.	8.	0.	-2.305937	2.983188	0.442406
17.	8.	0.	-4.267759	-1.290144	-0.051477
18.	8.	0.	-1.880067	-2.461286	-0.500298
19.	6.	0.	-1.550169	-3.238427	0.664512
20.	1.	0.	-4.275882	1.294818	0.376803
21.	1.	0.	0.685848	-1.860599	-0.833713
22.	1.	0.	3.069752	-1.405156	-1.238630
23.	1.	0.	3.412919	0.265254	-1.723450
24.	1.	0.	3.877194	0.831560	0.672042
25.	1.	0.	4.007777	-0.972956	2.098309
26.	1.	0.	5.539639	-1.494693	-0.452560
27.	1.	0.	6.073393	-0.378023	0.816142
28.	1.	0.	5.789216	0.228865	-0.819369
29.	1.	0.	-1.402585	3.384655	0.396049
30.	1.	0.	-3.970488	-2.167763	-0.348015
31.	1.	0.	-2.272419	-3.066470	1.471500
32.	1.	0.	-0.545812	-2.996317	1.027387
33.	1.	0.	-1.584950	-4.285505	0.357024

Conformer **2-2**

**2-2**		Standard Orientation (Ångstroms)			
No.	Atom	Type	X	Y	Z
1.	6.	0.	-3.309072	0.744343	0.222202
2.	6.	0.	-3.105855	-0.610772	-0.037784
3.	6.	0.	-1.817989	-1.124457	-0.285589
4.	6.	0.	-0.719210	-0.269460	-0.320456
5.	6.	0.	-0.914821	1.114031	-0.054150
6.	6.	0.	-2.218022	1.615164	0.227558

7.	6.	0.	0.620700	-0.709818	-0.629339
8.	6.	0.	1.641159	0.174616	-0.654930
9.	8.	0.	1.450145	1.503707	-0.363356
10.	6.	0.	0.204921	2.023513	-0.069879
11.	6.	0.	3.080670	-0.132770	-0.921192
12.	6.	0.	3.917800	-0.216269	0.379860
13.	8.	0.	3.466069	-1.289148	1.200274
14.	6.	0.	5.388377	-0.475254	0.084236
15.	8.	0.	0.150895	3.229058	0.157798
16.	8.	0.	-2.431058	2.911273	0.488048
17.	8.	0.	-4.164836	-1.450929	-0.057176
18.	8.	0.	-1.719731	-2.487157	-0.540765
19.	6.	0.	-1.364984	-3.273249	0.610139
20.	1.	0.	-4.306176	1.121288	0.413367
21.	1.	0.	0.805621	-1.744369	-0.888135
22.	1.	0.	3.154470	-1.092880	-1.440858
23.	1.	0.	3.495712	0.645570	-1.571401
24.	1.	0.	3.817084	0.742390	0.912663
25.	1.	0.	2.534016	-1.130671	1.405110
26.	1.	0.	5.506808	-1.414383	-0.465855
27.	1.	0.	5.948112	-0.555349	1.019505
28.	1.	0.	5.815836	0.337338	-0.510708
29.	1.	0.	-1.553626	3.365223	0.438100
30.	1.	0.	-3.828278	-2.305937	-0.376553
31.	1.	0.	-2.097162	-3.147523	1.416494
32.	1.	0.	-0.372697	-2.998552	0.983783
33.	1.	0.	-1.353633	-4.314268	0.281832

Conformer **2-3**

**2-3**		Standard Orientation (Ångstroms)			
No.	Atom	Type	X	Y	Z
1.	6.	0.	-3.211402	0.994398	0.393357
2.	6.	0.	-3.130419	-0.398165	0.416515
3.	6.	0.	-1.917656	-1.066249	0.163875
4.	6.	0.	-0.752801	-0.339465	-0.073844
5.	6.	0.	-0.826112	1.080675	-0.105483
6.	6.	0.	-2.066032	1.743936	0.121728
7.	6.	0.	0.538103	-0.950407	-0.276246
8.	6.	0.	1.621612	-0.185322	-0.512993
9.	8.	0.	1.537920	1.184597	-0.580491
10.	6.	0.	0.357046	1.862648	-0.366260

11.	6.	0.	3.025153	-0.658456	-0.729798
12.	6.	0.	3.998062	-0.209338	0.372185
13.	8.	0.	3.539999	-0.826342	1.574976
14.	6.	0.	5.438758	-0.609335	0.053573
15.	8.	0.	0.406659	3.089820	-0.422412
16.	8.	0.	-2.161986	3.080117	0.093207
17.	8.	0.	-4.237650	-1.125348	0.691205
18.	8.	0.	-1.941474	-2.455870	0.215208
19.	6.	0.	-2.070220	-3.082795	-1.071905
20.	1.	0.	-4.153388	1.491556	0.589073
21.	1.	0.	0.648581	-2.023833	-0.199036
22.	1.	0.	3.029292	-1.751669	-0.778914
23.	1.	0.	3.385717	-0.274292	-1.692010
24.	1.	0.	3.934712	0.885986	0.457351
25.	1.	0.	4.065215	-0.477262	2.306643
26.	1.	0.	5.519322	-1.695506	-0.057174
27.	1.	0.	6.110923	-0.300915	0.862090
28.	1.	0.	5.790917	-0.134325	-0.868117
29.	1.	0.	-1.256630	3.429625	-0.100680
30.	1.	0.	-3.939653	-2.047352	0.778126
31.	1.	0.	-1.213569	-2.846440	-1.712098
32.	1.	0.	-2.990392	-2.765195	-1.576831
33.	1.	0.	-2.103333	-4.158926	-0.890182

Conformer **2-4**

**2 -4**		Standard Orientation (Ångstroms)			
No.	Atom	Type	X	Y	Z
1.	6.	0.	-3.228624	0.843853	0.445431
2.	6.	0.	-3.055314	-0.539817	0.421852
3.	6.	0.	-1.805256	-1.117000	0.125186
4.	6.	0.	-0.698082	-0.305198	-0.108837
5.	6.	0.	-0.864303	1.107312	-0.089616
6.	6.	0.	-2.141344	1.677962	0.179358
7.	6.	0.	0.627312	-0.821090	-0.357396
8.	6.	0.	1.655150	0.022858	-0.593170
9.	8.	0.	1.482849	1.385953	-0.592676
10.	6.	0.	0.259616	1.975592	-0.342530
11.	6.	0.	3.087401	-0.354199	-0.803303
12.	6.	0.	3.928335	-0.230577	0.492831
13.	8.	0.	3.474307	-1.152147	1.477467

14.	6.	0.	5.396394	-0.544477	0.239775
15.	8.	0.	0.226439	3.203154	-0.357678
16.	8.	0.	-2.328401	3.003804	0.197641
17.	8.	0.	-4.105335	-1.346965	0.693459
18.	8.	0.	-1.732107	-2.504876	0.132937
19.	6.	0.	-1.861782	-3.103451	-1.168700
20.	1.	0.	-4.197612	1.270264	0.673626
21.	1.	0.	0.806387	-1.888456	-0.346641
22.	1.	0.	3.142551	-1.390703	-1.150082
23.	1.	0.	3.513439	0.293340	-1.577634
24.	1.	0.	3.836697	0.803268	0.862066
25.	1.	0.	2.547284	-0.952375	1.669154
26.	1.	0.	5.507205	-1.562611	-0.147032
27.	1.	0.	5.958127	-0.472804	1.174505
28.	1.	0.	5.827398	0.155591	-0.482065
29.	1.	0.	-1.454289	3.422765	0.000420
30.	1.	0.	-3.747396	-2.249793	0.748937
31.	1.	0.	-1.046538	-2.789319	-1.829129
32.	1.	0.	-2.818961	-2.837546	-1.632616
33.	1.	0.	-1.813336	-4.183604	-1.018005

Conformer **2-5**

**2-5**		Standard Orientation (Ångstroms)			
No.	Atom	Type	X	Y	Z
1.	6.	0.	3.193720	0.806948	0.286108
2.	6.	0.	3.026753	-0.556819	0.041435
3.	6.	0.	1.763470	-1.097412	-0.262791
4.	6.	0.	0.650907	-0.264535	-0.363468
5.	6.	0.	0.808378	1.125531	-0.105859
6.	6.	0.	2.087838	1.655959	0.227041
7.	6.	0.	-0.662165	-0.735467	-0.726320
8.	6.	0.	-1.701808	0.120246	-0.779328
9.	8.	0.	-1.550814	1.458680	-0.498420
10.	6.	0.	-0.329142	2.009228	-0.172508
11.	6.	0.	-3.121819	-0.229552	-1.109033
12.	6.	0.	-3.977259	-0.655002	0.101906
13.	8.	0.	-3.425499	-1.889825	0.561826
14.	6.	0.	-4.041073	0.384645	1.219738
15.	8.	0.	-0.308534	3.220061	0.040865
16.	8.	0.	2.262000	2.960626	0.478262

17.	8.	0.	4.099507	-1.378910	0.092132
18.	8.	0.	1.704787	-2.466621	-0.495862
19.	6.	0.	1.245280	-3.232339	0.631948
20.	1.	0.	4.173338	1.205044	0.519997
21.	1.	0.	-0.823914	-1.778206	-0.962457
22.	1.	0.	-3.595163	0.630041	-1.593900
23.	1.	0.	-3.125274	-1.062001	-1.818578
24.	1.	0.	-4.996465	-0.822972	-0.283677
25.	1.	0.	-3.911849	-2.152996	1.353967
26.	1.	0.	-3.050440	0.558540	1.647817
27.	1.	0.	-4.705229	0.040736	2.021490
28.	1.	0.	-4.429603	1.339490	0.853050
29.	1.	0.	1.376529	3.392452	0.383273
30.	1.	0.	3.786692	-2.248142	-0.213369
31.	1.	0.	0.219313	-2.960195	0.900961
32.	1.	0.	1.895987	-3.082522	1.501829
33.	1.	0.	1.276006	-4.279959	0.325962

Conformer **2-6**

**2-6**		Standard Orientation (Ångstroms)			
No.	Atom	Type	X	Y	Z
1.	6.	0.	-3.179466	1.182872	-0.295724
2.	6.	0.	-3.228644	-0.208936	-0.375370
3.	6.	0.	-2.070653	-0.994439	-0.214263
4.	6.	0.	-0.834094	-0.386183	-0.013591
5.	6.	0.	-0.774509	1.032805	0.075983
6.	6.	0.	-1.957705	1.814530	-0.060184
7.	6.	0.	0.402624	-1.122217	0.094422
8.	6.	0.	1.566864	-0.478138	0.311818
9.	8.	0.	1.609114	0.895156	0.430768
10.	6.	0.	0.485584	1.692840	0.296970
11.	6.	0.	2.925970	-1.091681	0.439760
12.	6.	0.	3.984686	-0.506928	-0.531925
13.	8.	0.	4.406846	0.785939	-0.127173
14.	6.	0.	5.233406	-1.380335	-0.571583
15.	8.	0.	0.663865	2.904406	0.382527
16.	8.	0.	-1.930471	3.151272	0.019904
17.	8.	0.	-4.409684	-0.820050	-0.617482
18.	8.	0.	-2.223255	-2.372107	-0.316949
19.	6.	0.	-2.357664	-3.038672	0.950817
20.	1.	0.	-4.080106	1.770662	-0.422562

21.	1.	0.	0.400766	-2.198189	-0.021024
22.	1.	0.	3.305563	-0.940236	1.459156
23.	1.	0.	2.824370	-2.169519	0.279727
24.	1.	0.	3.541346	-0.472046	-1.541896
25.	1.	0.	3.621041	1.343325	-0.035394
26.	1.	0.	5.659543	-1.481011	0.431830
27.	1.	0.	5.985552	-0.915592	-1.213994
28.	1.	0.	5.007262	-2.377471	-0.961551
29.	1.	0.	-0.990937	3.416855	0.170887
30.	1.	0.	-4.205537	-1.760815	-0.757839
31.	1.	0.	-3.224186	-2.660859	1.506203
32.	1.	0.	-1.458270	-2.906447	1.561533
33.	1.	0.	-2.496119	-4.098675	0.729382

Conformer **2-7**

**2-7**		Standard Orientation (Ångstroms)			
No.	Atom	Type	X	Y	Z
1.	6.	0.	3.239087	1.103898	0.114074
2.	6.	0.	3.259069	-0.280951	-0.052124
3.	6.	0.	2.069162	-1.018963	-0.204902
4.	6.	0.	0.839641	-0.365891	-0.239775
5.	6.	0.	0.808588	1.046536	-0.069149
6.	6.	0.	2.016830	1.777182	0.120115
7.	6.	0.	-0.413880	-1.047995	-0.454811
8.	6.	0.	-1.572535	-0.359396	-0.484346
9.	8.	0.	-1.594664	1.005781	-0.290316
10.	6.	0.	-0.447086	1.750628	-0.079069
11.	6.	0.	-2.944756	-0.913939	-0.706256
12.	6.	0.	-3.960815	-0.564054	0.412713
13.	8.	0.	-4.372766	0.791681	0.339463
14.	6.	0.	-5.222985	-1.410294	0.293033
15.	8.	0.	-0.602414	2.957256	0.084709
16.	8.	0.	2.015628	3.105431	0.292533
17.	8.	0.	4.443760	-0.931438	-0.070853
18.	8.	0.	2.194848	-2.393724	-0.366291
19.	6.	0.	2.029613	-3.140761	0.851847
20.	1.	0.	4.163949	1.654321	0.234002
21.	1.	0.	-0.423048	-2.116138	-0.628216
22.	1.	0.	-3.353478	-0.524779	-1.648278
23.	1.	0.	-2.856118	-2.000098	-0.806108
24.	1.	0.	-3.483310	-0.772124	1.385996

25.	1.	0.	-3.580224	1.347132	0.341078
26.	1.	0.	-5.686068	-1.267121	-0.688588
27.	1.	0.	-5.943529	-1.103465	1.055344
28.	1.	0.	-5.001577	-2.473508	0.427737
29.	1.	0.	1.074171	3.403737	0.263228
30.	1.	0.	4.245507	-1.850809	-0.319411
31.	1.	0.	1.025228	-3.000820	1.265575
32.	1.	0.	2.769425	-2.841016	1.603490
33.	1.	0.	2.174321	-4.191202	0.592091

Table S5. Gibbs free energiesa and equilibrium populationsb of low-energy conformers of cytospomarin (**2**)

Conformers	In MeOH	
	Boltzmann population (%)	*OR*
**2-1**	11.53	322.56
**2-2**	17.39	349.9
**2-3**	7.98	22.87
**2-4**	20.22	66.1
**2-5**	1.64	-251.39
**2-6**	21.89	95.84
**2-7**	19.34	-209.57
**Average**		89.55

Table S6. Cartesian coordinates for the low-energy reoptimized MMFF conformers of cytospomarin (**2**) at B3LYP/6-31+G(d,p) level of theory in CH_3OH

Conformer **2-1**

**2-1**		Standard Orientation (Ångstroms)			
No.	Atom	Type	X	Y	Z
1.	6.	0.	-3.299084	0.862809	0.197751
2.	6.	0.	-3.165693	-0.505707	-0.039249
3.	6.	0.	-1.906543	-1.090102	-0.271458
4.	6.	0.	-0.762265	-0.296083	-0.311974
5.	6.	0.	-0.886472	1.099096	-0.064602
6.	6.	0.	-2.162893	1.673068	0.200317
7.	6.	0.	0.552173	-0.811693	-0.605633
8.	6.	0.	1.617871	0.012279	-0.617353
9.	8.	0.	1.496522	1.354960	-0.351341
10.	6.	0.	0.281362	1.944947	-0.076026
11.	6.	0.	3.037622	-0.362971	-0.906492
12.	6.	0.	3.970640	-0.201353	0.304045

13.	8.	0.	3.499593	-1.120947	1.290228
14.	6.	0.	5.428422	-0.477193	-0.064162
15.	8.	0.	0.290969	3.155519	0.138284
16.	8.	0.	-2.305937	2.983188	0.442406
17.	8.	0.	-4.267759	-1.290144	-0.051477
18.	8.	0.	-1.880067	-2.461286	-0.500298
19.	6.	0.	-1.550169	-3.238427	0.664512
20.	1.	0.	-4.275882	1.294818	0.376803
21.	1.	0.	0.685848	-1.860599	-0.833713
22.	1.	0.	3.069752	-1.405156	-1.238630
23.	1.	0.	3.412919	0.265254	-1.723450
24.	1.	0.	3.877194	0.831560	0.672042
25.	1.	0.	4.007777	-0.972956	2.098309
26.	1.	0.	5.539639	-1.494693	-0.452560
27.	1.	0.	6.073393	-0.378023	0.816142
28.	1.	0.	5.789216	0.228865	-0.819369
29.	1.	0.	-1.402585	3.384655	0.396049
30.	1.	0.	-3.970488	-2.167763	-0.348015
31.	1.	0.	-2.272419	-3.066470	1.471500
32.	1.	0.	-0.545812	-2.996317	1.027387
33.	1.	0.	-1.584950	-4.285505	0.357024

Conformer **2-2**

**2-2**		Standard Orientation (Ångstroms)			
No.	Atom	Type	X	Y	Z
1.	6.	0.	-3.309072	0.744343	0.222202
2.	6.	0.	-3.105855	-0.610772	-0.037784
3.	6.	0.	-1.817989	-1.124457	-0.285589
4.	6.	0.	-0.719210	-0.269460	-0.320456
5.	6.	0.	-0.914821	1.114031	-0.054150
6.	6.	0.	-2.218022	1.615164	0.227558
7.	6.	0.	0.620700	-0.709818	-0.629339
8.	6.	0.	1.641159	0.174616	-0.654930
9.	8.	0.	1.450145	1.503707	-0.363356
10.	6.	0.	0.204921	2.023513	-0.069879
11.	6.	0.	3.080670	-0.132770	-0.921192
12.	6.	0.	3.917800	-0.216269	0.379860
13.	8.	0.	3.466069	-1.289148	1.200274
14.	6.	0.	5.388377	-0.475254	0.084236

15.	8.	0.	0.150895	3.229058	0.157798
16.	8.	0.	-2.431058	2.911273	0.488048
17.	8.	0.	-4.164836	-1.450929	-0.057176
18.	8.	0.	-1.719731	-2.487157	-0.540765
19.	6.	0.	-1.364984	-3.273249	0.610139
20.	1.	0.	-4.306176	1.121288	0.413367
21.	1.	0.	0.805621	-1.744369	-0.888135
22.	1.	0.	3.154470	-1.092880	-1.440858
23.	1.	0.	3.495712	0.645570	-1.571401
24.	1.	0.	3.817084	0.742390	0.912663
25.	1.	0.	2.534016	-1.130671	1.405110
26.	1.	0.	5.506808	-1.414383	-0.465855
27.	1.	0.	5.948112	-0.555349	1.019505
28.	1.	0.	5.815836	0.337338	-0.510708
29.	1.	0.	-1.553626	3.365223	0.438100
30.	1.	0.	-3.828278	-2.305937	-0.376553
31.	1.	0.	-2.097162	-3.147523	1.416494
32.	1.	0.	-0.372697	-2.998552	0.983783
33.	1.	0.	-1.353633	-4.314268	0.281832

Conformer **2-3**

**2-3**		Standard Orientation (Ångstroms)			
No.	Atom	Type	X	Y	Z
1.	6.	0.	-3.211402	0.994398	0.393357
2.	6.	0.	-3.130419	-0.398165	0.416515
3.	6.	0.	-1.917656	-1.066249	0.163875
4.	6.	0.	-0.752801	-0.339465	-0.073844
5.	6.	0.	-0.826112	1.080675	-0.105483
6.	6.	0.	-2.066032	1.743936	0.121728
7.	6.	0.	0.538103	-0.950407	-0.276246
8.	6.	0.	1.621612	-0.185322	-0.512993
9.	8.	0.	1.537920	1.184597	-0.580491
10.	6.	0.	0.357046	1.862648	-0.366260
11.	6.	0.	3.025153	-0.658456	-0.729798
12.	6.	0.	3.998062	-0.209338	0.372185
13.	8.	0.	3.539999	-0.826342	1.574976
14.	6.	0.	5.438758	-0.609335	0.053573
15.	8.	0.	0.406659	3.089820	-0.422412
16.	8.	0.	-2.161986	3.080117	0.093207
17.	8.	0.	-4.237650	-1.125348	0.691205

18.	8.	0.	-1.941474	-2.455870	0.215208
19.	6.	0.	-2.070220	-3.082795	-1.071905
20.	1.	0.	-4.153388	1.491556	0.589073
21.	1.	0.	0.648581	-2.023833	-0.199036
22.	1.	0.	3.029292	-1.751669	-0.778914
23.	1.	0.	3.385717	-0.274292	-1.692010
24.	1.	0.	3.934712	0.885986	0.457351
25.	1.	0.	4.065215	-0.477262	2.306643
26.	1.	0.	5.519322	-1.695506	-0.057174
27.	1.	0.	6.110923	-0.300915	0.862090
28.	1.	0.	5.790917	-0.134325	-0.868117
29.	1.	0.	-1.256630	3.429625	-0.100680
30.	1.	0.	-3.939653	-2.047352	0.778126
31.	1.	0.	-1.213569	-2.846440	-1.712098
32.	1.	0.	-2.990392	-2.765195	-1.576831
33.	1.	0.	-2.103333	-4.158926	-0.890182

Conformer **2-4**

**2 -4**		Standard Orientation (Ångstroms)			
No.	Atom	Type	X	Y	Z
1.	6.	0.	-3.228624	0.843853	0.445431
2.	6.	0.	-3.055314	-0.539817	0.421852
3.	6.	0.	-1.805256	-1.117000	0.125186
4.	6.	0.	-0.698082	-0.305198	-0.108837
5.	6.	0.	-0.864303	1.107312	-0.089616
6.	6.	0.	-2.141344	1.677962	0.179358
7.	6.	0.	0.627312	-0.821090	-0.357396
8.	6.	0.	1.655150	0.022858	-0.593170
9.	8.	0.	1.482849	1.385953	-0.592676
10.	6.	0.	0.259616	1.975592	-0.342530
11.	6.	0.	3.087401	-0.354199	-0.803303
12.	6.	0.	3.928335	-0.230577	0.492831
13.	8.	0.	3.474307	-1.152147	1.477467
14.	6.	0.	5.396394	-0.544477	0.239775
15.	8.	0.	0.226439	3.203154	-0.357678
16.	8.	0.	-2.328401	3.003804	0.197641
17.	8.	0.	-4.105335	-1.346965	0.693459

18.	8.	0.	-1.732107	-2.504876	0.132937
19.	6.	0.	-1.861782	-3.103451	-1.168700
20.	1.	0.	-4.197612	1.270264	0.673626
21.	1.	0.	0.806387	-1.888456	-0.346641
22.	1.	0.	3.142551	-1.390703	-1.150082
23.	1.	0.	3.513439	0.293340	-1.577634
24.	1.	0.	3.836697	0.803268	0.862066
25.	1.	0.	2.547284	-0.952375	1.669154
26.	1.	0.	5.507205	-1.562611	-0.147032
27.	1.	0.	5.958127	-0.472804	1.174505
28.	1.	0.	5.827398	0.155591	-0.482065
29.	1.	0.	-1.454289	3.422765	0.000420
30.	1.	0.	-3.747396	-2.249793	0.748937
31.	1.	0.	-1.046538	-2.789319	-1.829129
32.	1.	0.	-2.818961	-2.837546	-1.632616
33.	1.	0.	-1.813336	-4.183604	-1.018005

Conformer **2-5**

**2-5**		Standard Orientation (Ångstroms)			
No.	Atom	Type	X	Y	Z
1.	6.	0.	3.193720	0.806948	0.286108
2.	6.	0.	3.026753	-0.556819	0.041435
3.	6.	0.	1.763470	-1.097412	-0.262791
4.	6.	0.	0.650907	-0.264535	-0.363468
5.	6.	0.	0.808378	1.125531	-0.105859
6.	6.	0.	2.087838	1.655959	0.227041
7.	6.	0.	-0.662165	-0.735467	-0.726320
8.	6.	0.	-1.701808	0.120246	-0.779328
9.	8.	0.	-1.550814	1.458680	-0.498420
10.	6.	0.	-0.329142	2.009228	-0.172508
11.	6.	0.	-3.121819	-0.229552	-1.109033
12.	6.	0.	-3.977259	-0.655002	0.101906
13.	8.	0.	-3.425499	-1.889825	0.561826
14.	6.	0.	-4.041073	0.384645	1.219738
15.	8.	0.	-0.308534	3.220061	0.040865
16.	8.	0.	2.262000	2.960626	0.478262
17.	8.	0.	4.099507	-1.378910	0.092132
18.	8.	0.	1.704787	-2.466621	-0.495862

19.	6.	0.	1.245280	-3.232339	0.631948
20.	1.	0.	4.173338	1.205044	0.519997
21.	1.	0.	-0.823914	-1.778206	-0.962457
22.	1.	0.	-3.595163	0.630041	-1.593900
23.	1.	0.	-3.125274	-1.062001	-1.818578
24.	1.	0.	-4.996465	-0.822972	-0.283677
25.	1.	0.	-3.911849	-2.152996	1.353967
26.	1.	0.	-3.050440	0.558540	1.647817
27.	1.	0.	-4.705229	0.040736	2.021490
28.	1.	0.	-4.429603	1.339490	0.853050
29.	1.	0.	1.376529	3.392452	0.383273
30.	1.	0.	3.786692	-2.248142	-0.213369
31.	1.	0.	0.219313	-2.960195	0.900961
32.	1.	0.	1.895987	-3.082522	1.501829
33.	1.	0.	1.276006	-4.279959	0.325962

Conformer **2-6**

**2-6**		Standard Orientation (Ångstroms)			
No.	Atom	Type	X	Y	Z
1.	6.	0.	-3.179466	1.182872	-0.295724
2.	6.	0.	-3.228644	-0.208936	-0.375370
3.	6.	0.	-2.070653	-0.994439	-0.214263
4.	6.	0.	-0.834094	-0.386183	-0.013591
5.	6.	0.	-0.774509	1.032805	0.075983
6.	6.	0.	-1.957705	1.814530	-0.060184
7.	6.	0.	0.402624	-1.122217	0.094422
8.	6.	0.	1.566864	-0.478138	0.311818
9.	8.	0.	1.609114	0.895156	0.430768
10.	6.	0.	0.485584	1.692840	0.296970
11.	6.	0.	2.925970	-1.091681	0.439760
12.	6.	0.	3.984686	-0.506928	-0.531925
13.	8.	0.	4.406846	0.785939	-0.127173
14.	6.	0.	5.233406	-1.380335	-0.571583
15.	8.	0.	0.663865	2.904406	0.382527
16.	8.	0.	-1.930471	3.151272	0.019904
17.	8.	0.	-4.409684	-0.820050	-0.617482
18.	8.	0.	-2.223255	-2.372107	-0.316949
19.	6.	0.	-2.357664	-3.038672	0.950817
20.	1.	0.	-4.080106	1.770662	-0.422562
21.	1.	0.	0.400766	-2.198189	-0.021024

22.	1.	0.	3.305563	-0.940236	1.459156
23.	1.	0.	2.824370	-2.169519	0.279727
24.	1.	0.	3.541346	-0.472046	-1.541896
25.	1.	0.	3.621041	1.343325	-0.035394
26.	1.	0.	5.659543	-1.481011	0.431830
27.	1.	0.	5.985552	-0.915592	-1.213994
28.	1.	0.	5.007262	-2.377471	-0.961551
29.	1.	0.	-0.990937	3.416855	0.170887
30.	1.	0.	-4.205537	-1.760815	-0.757839
31.	1.	0.	-3.224186	-2.660859	1.506203
32.	1.	0.	-1.458270	-2.906447	1.561533
33.	1.	0.	-2.496119	-4.098675	0.729382

Conformer **2-7**

**2-7**		Standard Orientation (Ångstroms)			
No.	Atom	Type	X	Y	Z
1.	6.	0.	3.239087	1.103898	0.114074
2.	6.	0.	3.259069	-0.280951	-0.052124
3.	6.	0.	2.069162	-1.018963	-0.204902
4.	6.	0.	0.839641	-0.365891	-0.239775
5.	6.	0.	0.808588	1.046536	-0.069149
6.	6.	0.	2.016830	1.777182	0.120115
7.	6.	0.	-0.413880	-1.047995	-0.454811
8.	6.	0.	-1.572535	-0.359396	-0.484346
9.	8.	0.	-1.594664	1.005781	-0.290316
10.	6.	0.	-0.447086	1.750628	-0.079069
11.	6.	0.	-2.944756	-0.913939	-0.706256
12.	6.	0.	-3.960815	-0.564054	0.412713
13.	8.	0.	-4.372766	0.791681	0.339463
14.	6.	0.	-5.222985	-1.410294	0.293033
15.	8.	0.	-0.602414	2.957256	0.084709
16.	8.	0.	2.015628	3.105431	0.292533
17.	8.	0.	4.443760	-0.931438	-0.070853
18.	8.	0.	2.194848	-2.393724	-0.366291
19.	6.	0.	2.029613	-3.140761	0.851847
20.	1.	0.	4.163949	1.654321	0.234002
21.	1.	0.	-0.423048	-2.116138	-0.628216
22.	1.	0.	-3.353478	-0.524779	-1.648278
23.	1.	0.	-2.856118	-2.000098	-0.806108
24.	1.	0.	-3.483310	-0.772124	1.385996
25.	1.	0.	-3.580224	1.347132	0.341078

26.	1.	0.	-5.686068	-1.267121	-0.688588
27.	1.	0.	-5.943529	-1.103465	1.055344
28.	1.	0.	-5.001577	-2.473508	0.427737
29.	1.	0.	1.074171	3.403737	0.263228
30.	1.	0.	4.245507	-1.850809	-0.319411
31.	1.	0.	1.025228	-3.000820	1.265575
32.	1.	0.	2.769425	-2.841016	1.603490
33.	1.	0.	2.174321	-4.191202	0.592091

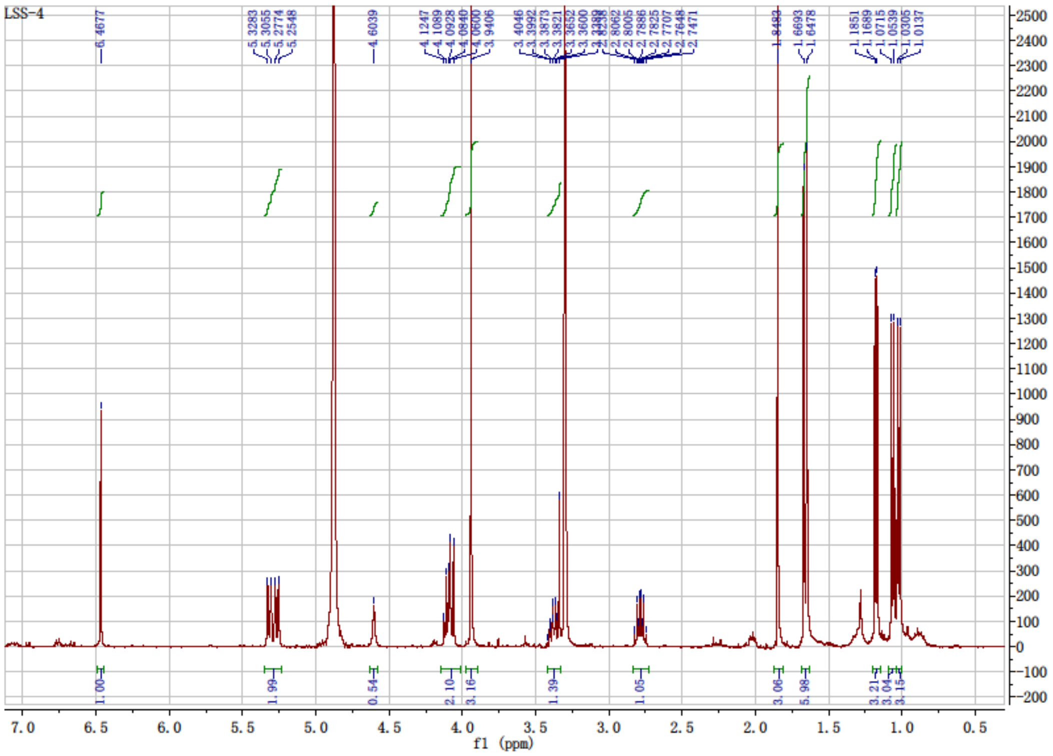

Figure S1. ^{1}H-NMR of cytospyrone (**1**) in CD_3OD

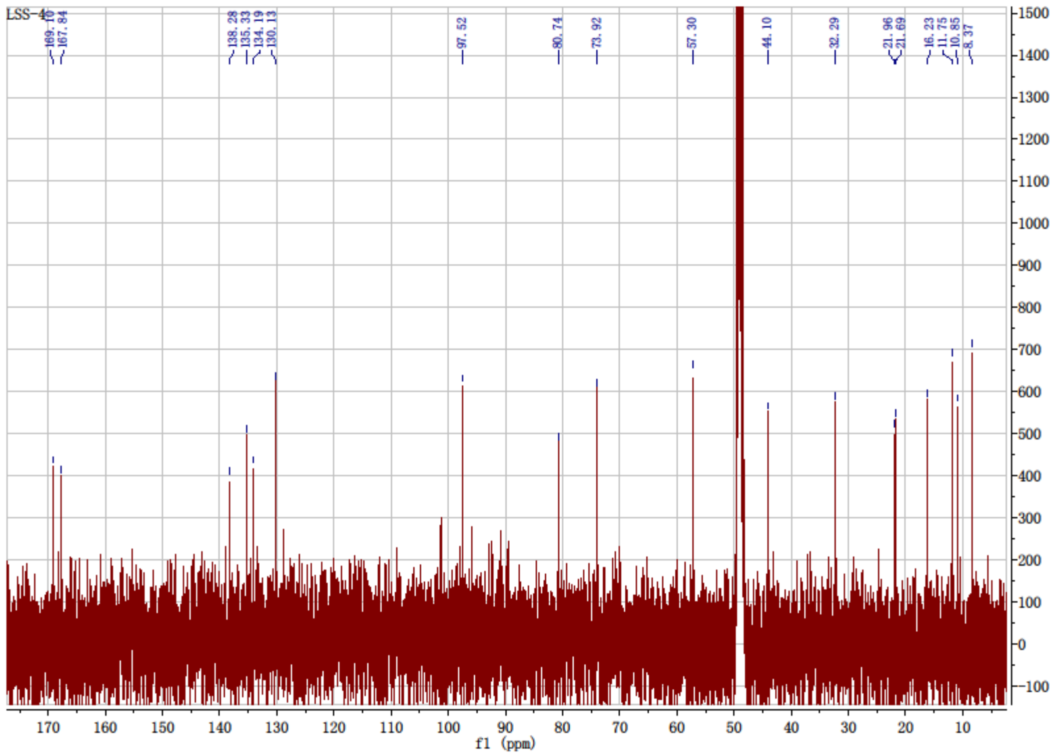

Figure S2. ^{13}C-NMR of cytospyrone (**1**) in CD_3OD

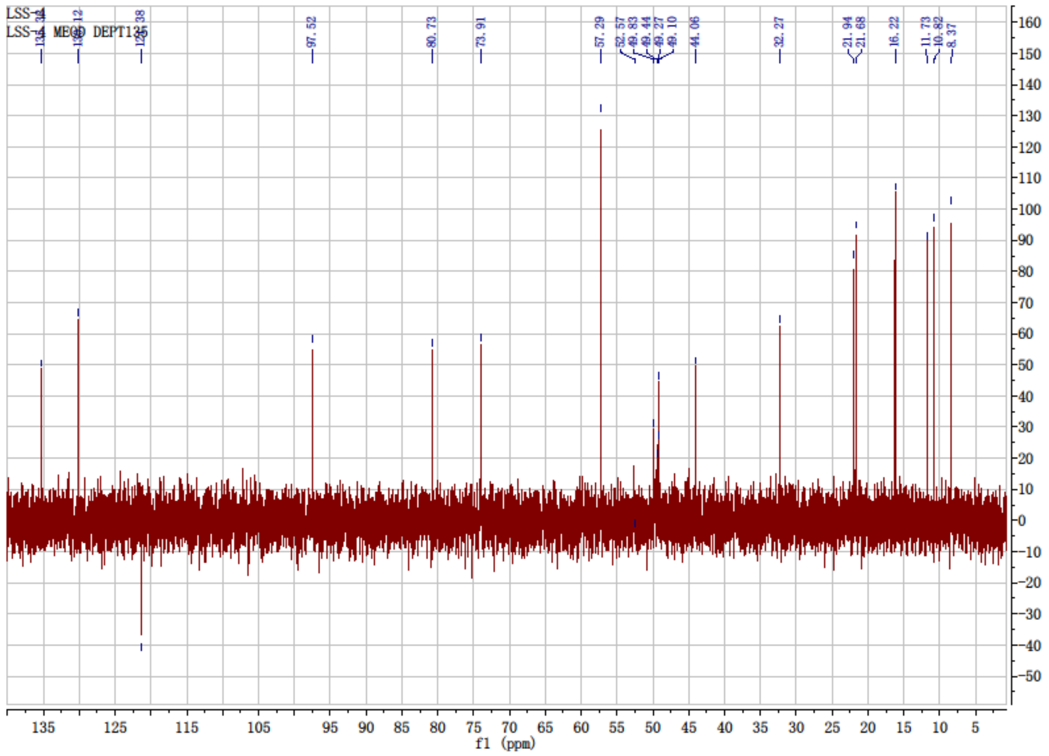

Figure S3. DEPT of cytospyrone (**1**) in CD_3OD

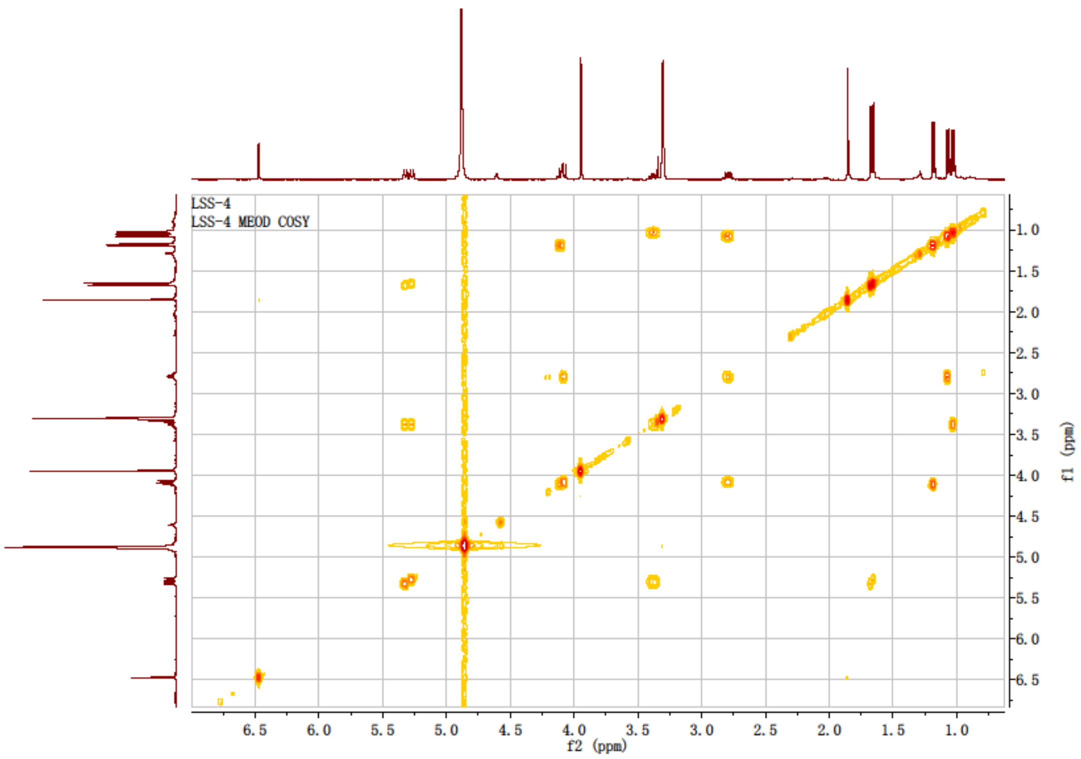

Figure S4. ^{1}H-^{1}H COSY of cytospyrone (**1**) in CD_3OD

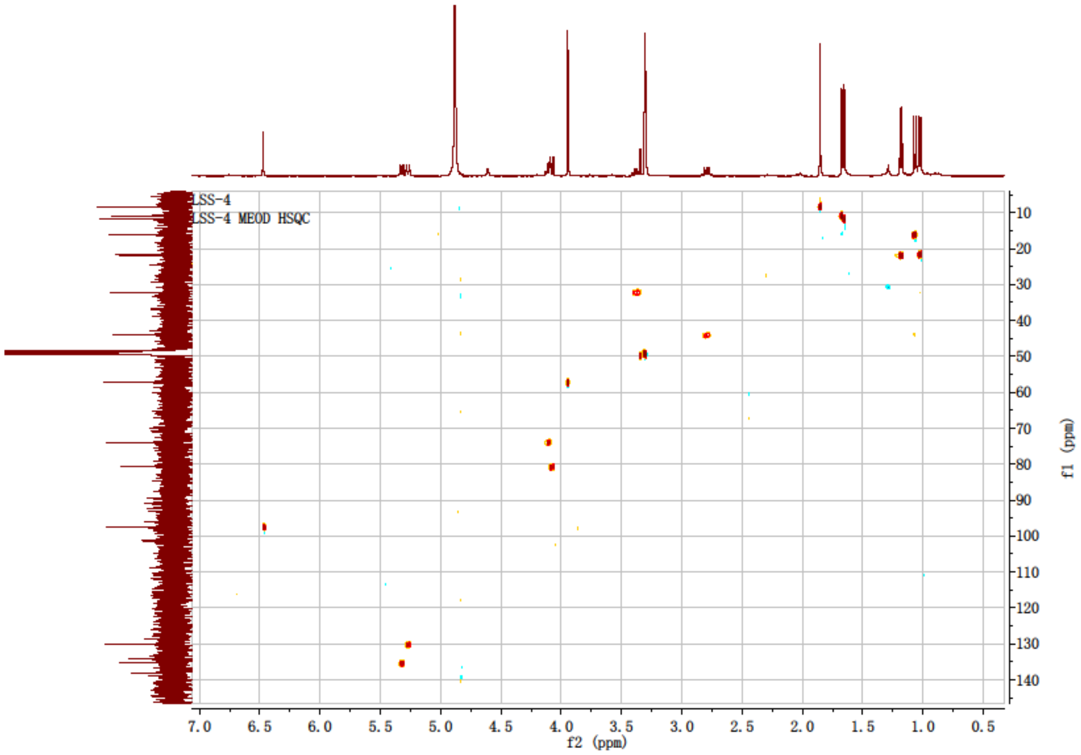

Figure S5. HMQC of cytospyrone (**1**) in CD_3OD

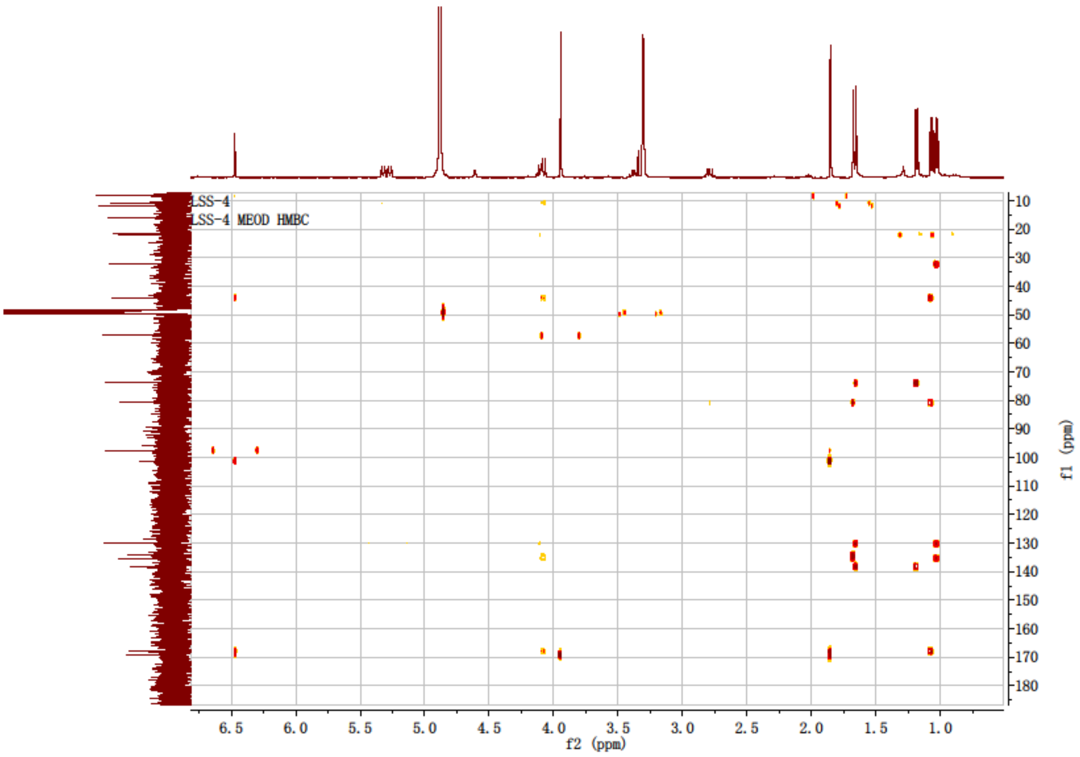

Figure S6. HMBC of cytospyrone (**1**) in CD_3OD

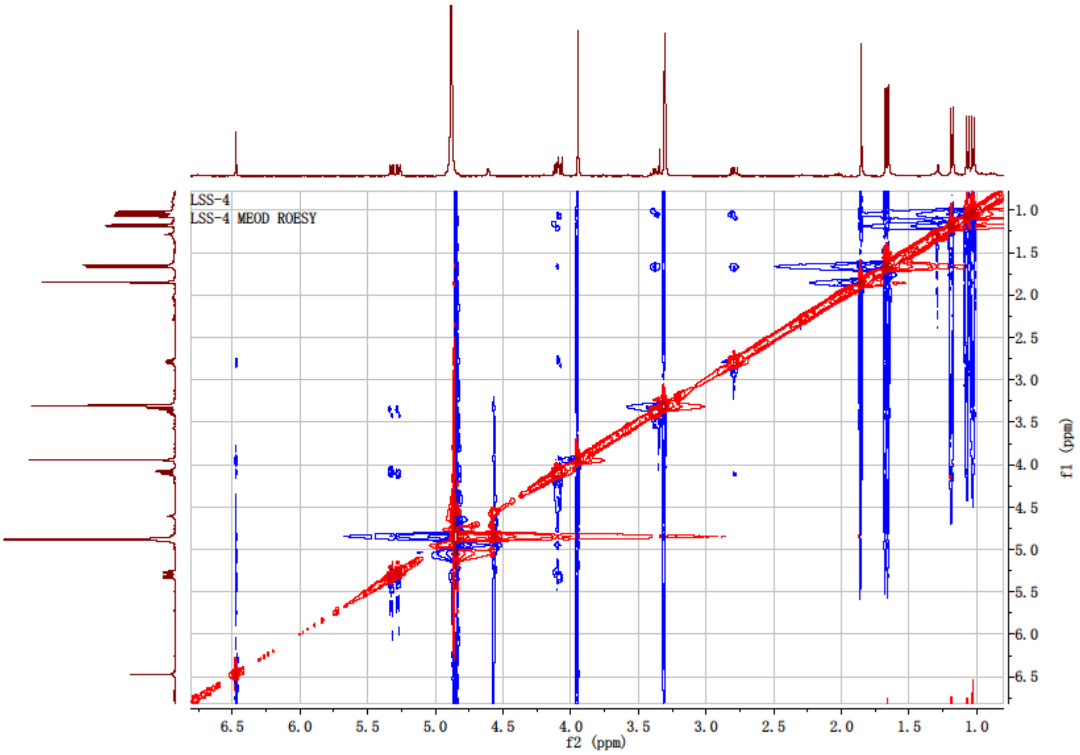

Figure S7. ROESY of cytospyrone (**1**) in CD_3OD

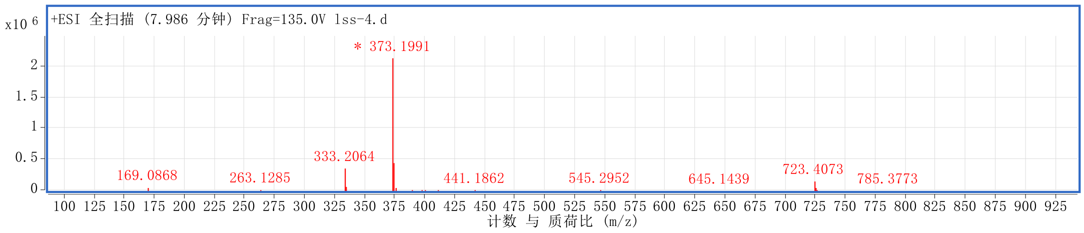

Figure S8. HR-ESI-MS of cytospyrone (**1**)

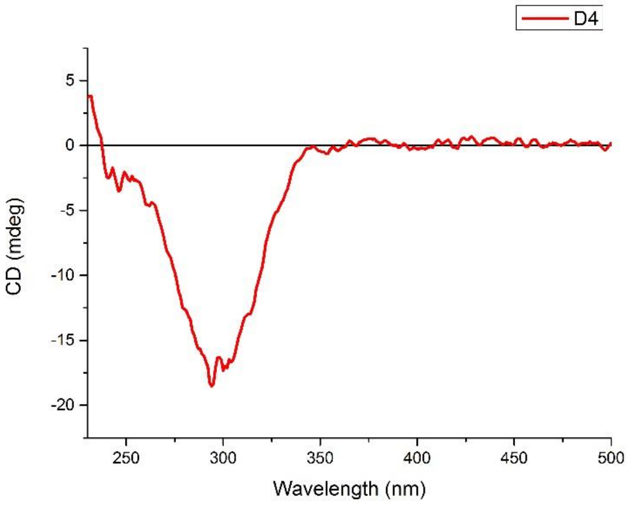

Figure S9 CD spectrum of cytospyrone (**1**)

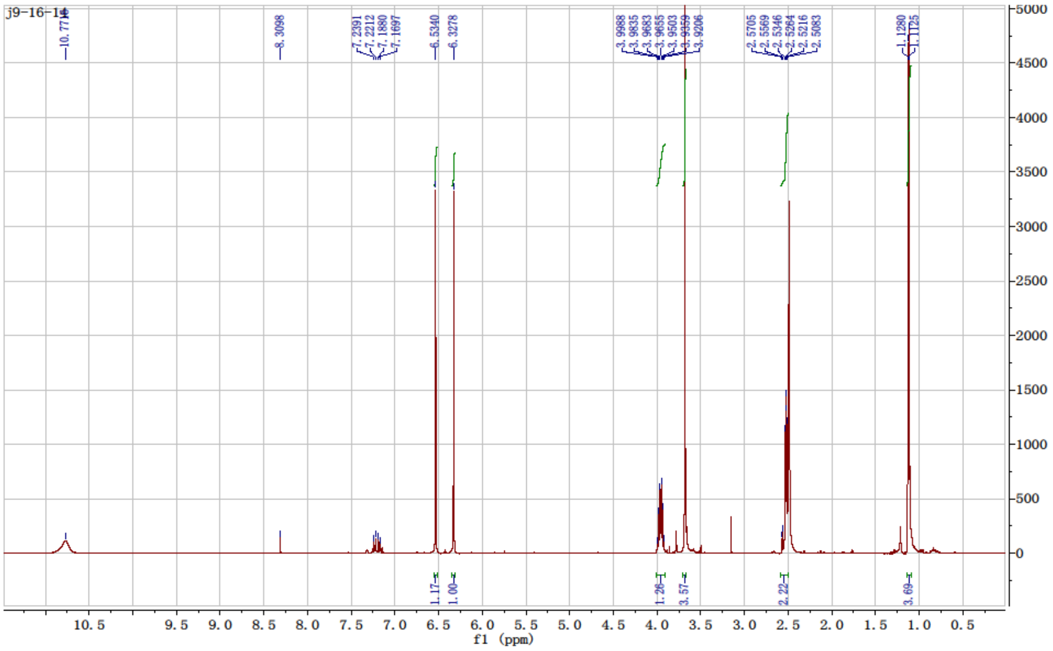

Figure S10. ^{1}H-NMR of cytospomarin (**2**) in DMSO-d_6

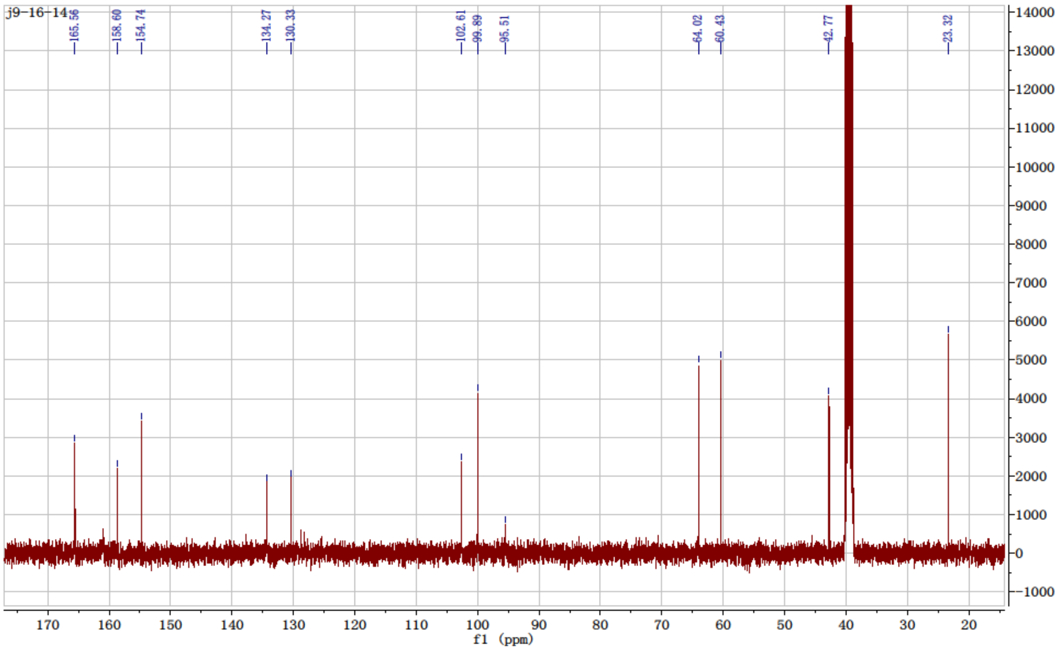

Figure S11. ^{13}C-NMR of cytospomarin (**2**) in DMSO-*d*6

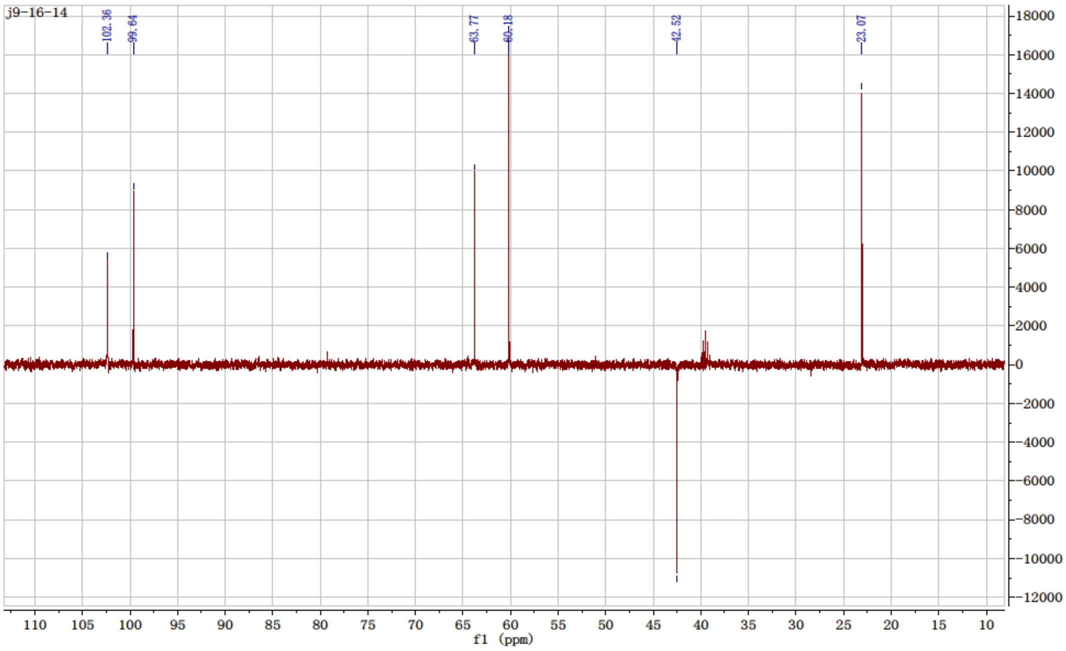

Figure S12. DEPT of cytospomarin (**2**) in DMSO-*d*6

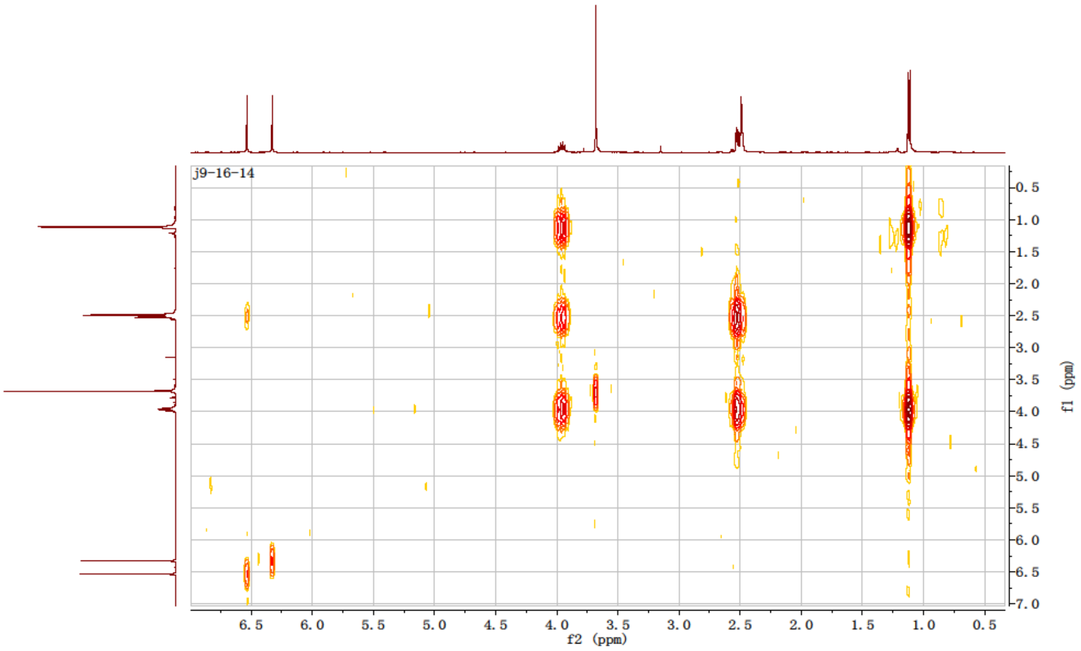

Figure S13. ^{1}H-^{1}H COSY of cytospomarin (**2**) in DMSO-*d*6

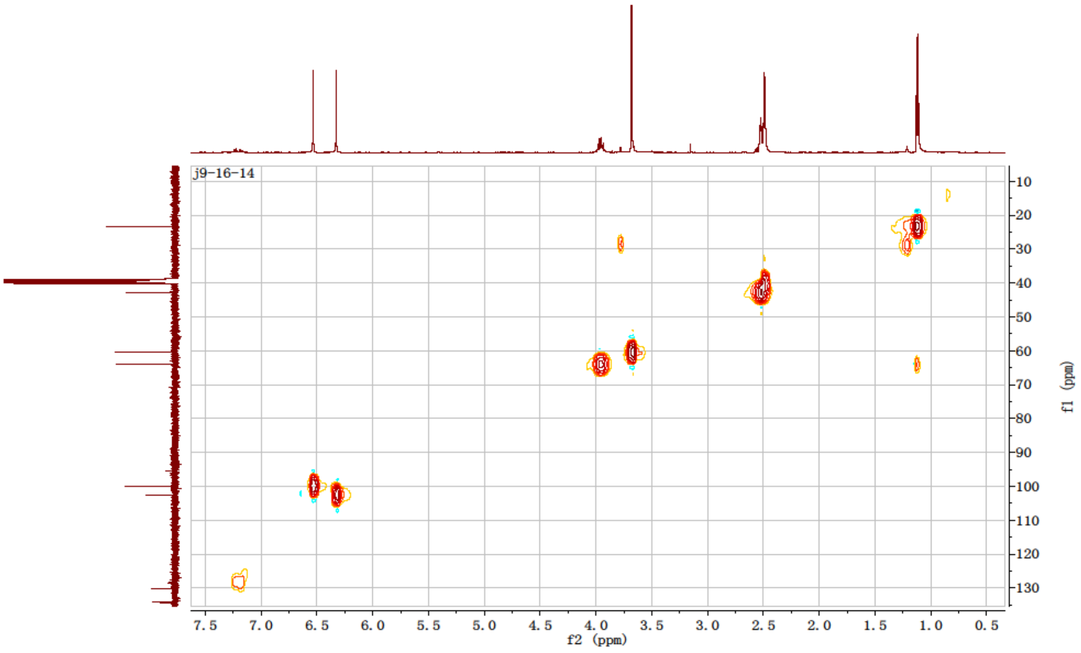

Figure S14. HMQC of cytospomarin (**2**) in DMSO-*d*6

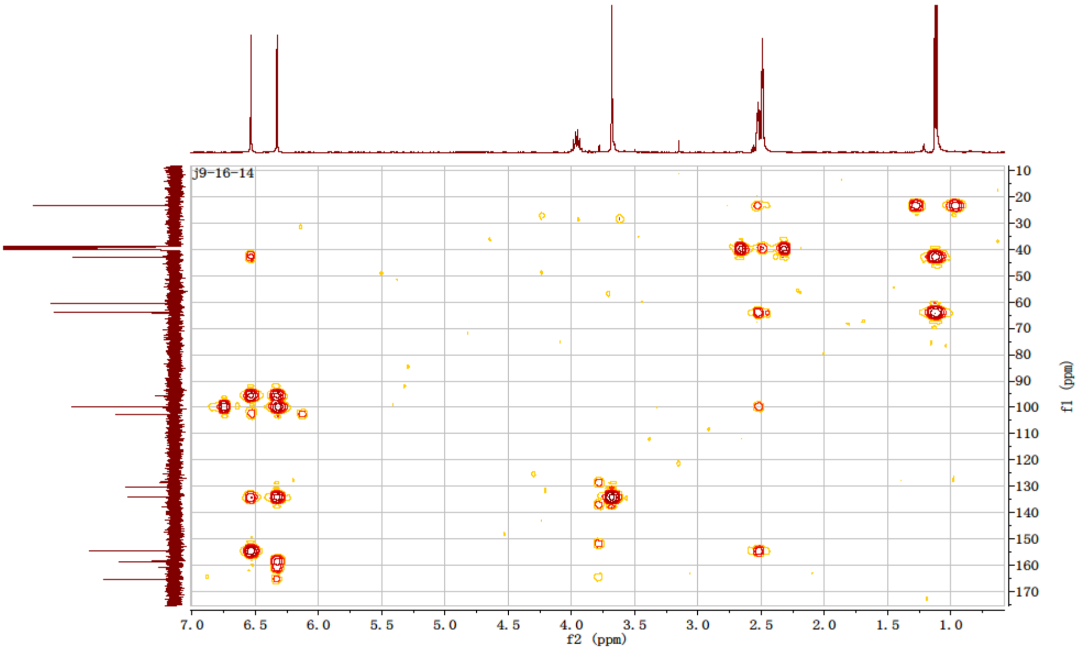

Figure S15. HMBC of cytospomarin (**2**) in DMSO-*d*6

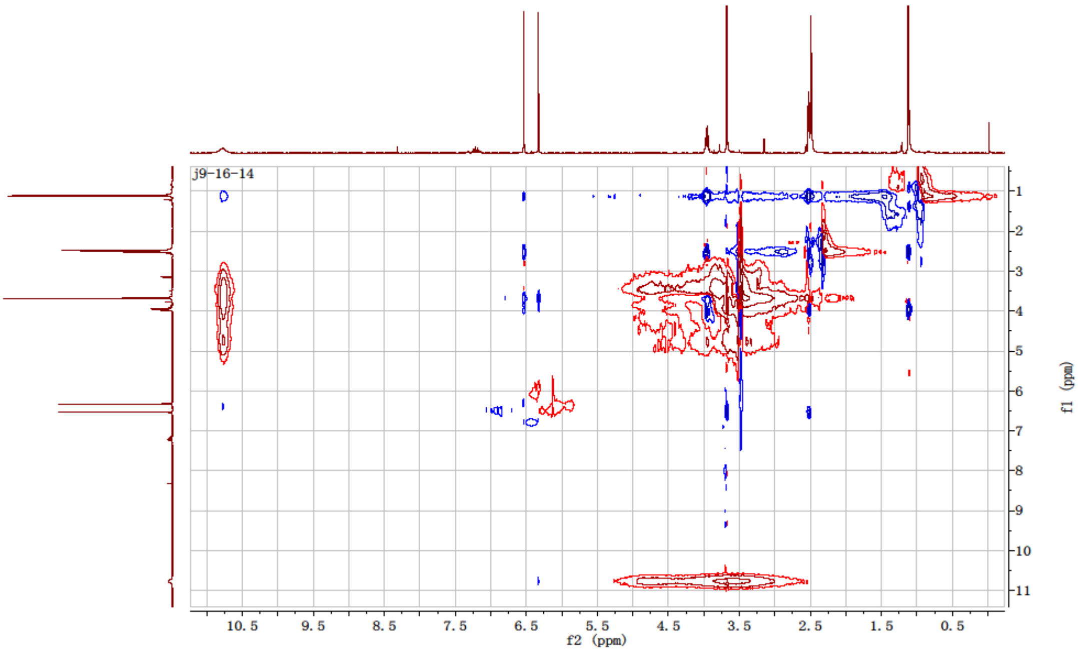

Figure S16. ROESY of cytospomarin (**2**) in DMSO-*d*6

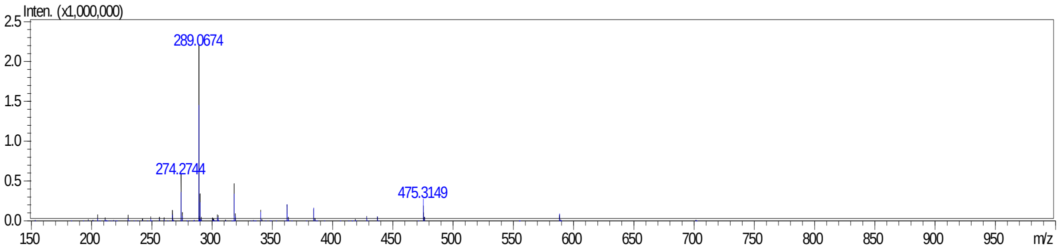

Figure S17. HR-ESI-MS of cytospomarin (**2**)

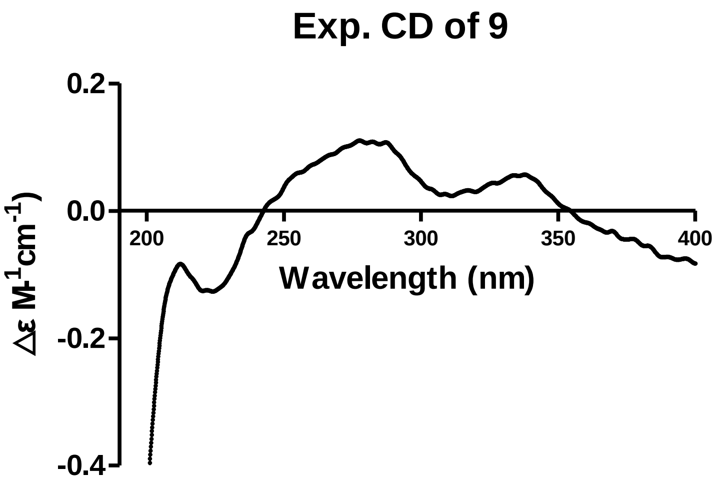

Figure S18. CD spectrum of cytospomarin (**2**)
